# Molecular disruption of DNA polymerase β for platinum sensitisation and synthetic lethality in epithelial ovarian cancers

**DOI:** 10.1038/s41388-021-01710-y

**Published:** 2021-03-05

**Authors:** Reem Ali, Adel Alblihy, Islam M. Miligy, Muslim L. Alabdullah, Mansour Alsaleem, Michael S. Toss, Mashael Algethami, Tarek Abdel-Fatah, Paul Moseley, Stephen Chan, Nigel P. Mongan, Satya Narayan, Emad A. Rakha, Srinivasan Madhusudan

**Affiliations:** 1 Translational Oncology, Division of Cancer & Stem Cells, School of Medicine, University of Nottingham, Nottingham, UK; 2 Medical Center, King Fahad Security College (KFSC), Riyadh, Saudi Arabia; 3 Academic Pathology, Division of Cancer & Stem Cells, School of Medicine, University of Nottingham, Nottingham, UK; 4 Department of Pathology, Faculty of Medicine, Menoufia University, Menoufia, Egypt; 5 Department of Oncology, Nottingham University Hospitals, Nottingham, UK; 6 Faculty of medicine and Health Sciences, Centre for Cancer Sciences, University of Nottingham, Sutton Bonington Campus, Sutton Bonington, Leicestershire UK; 7 Department of Pharmacology, Weill Cornell Medicine, New York, NY USA; 8 Department of Anatomy and Cell Biology, College of Medicine, University of Florida, Gainesville, FL USA

**Keywords:** Ovarian cancer, Predictive markers

## Abstract

Targeting PARP1 [Poly(ADP-Ribose) Polymerase 1] for synthetic lethality is a new strategy for *BRCA* germ-line mutated or platinum sensitive ovarian cancers. However, not all patients respond due to intrinsic or acquired resistance to PARP1 inhibitor. Development of alternative synthetic lethality approaches is a high priority. DNA polymerase β (Polβ), a critical player in base excision repair (BER), interacts with PARP1 during DNA repair. Here we show that polβ deficiency is a predictor of platinum sensitivity in human ovarian tumours. Polβ depletion not only increased platinum sensitivity but also reduced invasion, migration and impaired EMT (epithelial to mesenchymal transition) of ovarian cancer cells. Polβ small molecular inhibitors (Pamoic acid and NSC666719) were selectively toxic to BRCA2 deficient cells and associated with double-strand breaks (DSB) accumulation, cell cycle arrest and increased apoptosis. Interestingly, PARG [Poly(ADP-Ribose) Glycohydrolase] inhibitor (PDD00017273) [but not PARP1 inhibitor (Olaparib)] was synthetically lethal in polβ deficient cells. Selective toxicity to PDD00017273 was associated with poly (ADP-ribose) accumulation, reduced nicotinamide adenine dinucleotide (NAD^+^) level, DSB accumulation, cell cycle arrest and increased apoptosis. In human tumours, polβ-PARG co-expression adversely impacted survival in patients. Our data provide evidence that polβ targeting is a novel strategy and warrants further pharmaceutical development in epithelial ovarian cancers.

## Introduction

In BRCA germ-line deficient and platinum sensitive sporadic epithelial ovarian cancers, PARP inhibitor (Niraparib, Olaparib, Rucaparib) maintenance therapy improves progression-free survival (PFS) [1–3]. However, not all patients respond [4] either due to intrinsic resistance or acquired resistance to PARP inhibitors. Therefore, the development of alternative DNA repair targets and synthetic lethality approaches is required.

Base excision repair (BER) is essential for the removal of bases damaged by alkylation, oxidation or deamination [5, 6]. BER is performed by at least two major sub-pathways: the short-patch pathway (SP-BER) and long-patch pathway (LP-BER) [7]. Both pathways are initiated by a damage-specific DNA glycosylase, which removes the damaged base creating an abasic site. APE1 then cleaves the phosphodiester bond 5′ to the AP site thereby generating a nick with 5′-sugar phosphate (dRP) and 3′-hydroxyl group. DNA polymerase β (polβ) adds the first nucleotide to the 3′-end of the incised AP site. Normally, the reaction continues through the short-patch repair pathway where polβ removes the 5′-sugar phosphate residue by the process of β-elimination [8] and DNA ligase III-XRCC1 heterodimer (or DNA ligase I) then completes the repair [9, 10]. Polβ-mediated lyase activity is the rate-limiting step in SP-BER. The processing of oxidised AP sites generates a 5′ residue that is resistant to β-elimination (mediated by polβ) and therefore requires additional DNA synthesis via LP-BER. A role for the polymerase activity of Pol β under the coordination of the Rad9-Rad1-Hus1 sliding clamp complex (9-1-1 complex) in LP-BER has also been described [10].

Polβ is a key player in BER [11]. Polβ interacts with several components of the BER machinery including XRCC1, ligase III and PARP1 to accomplish its biochemical functions [11]. In the current study, we hypothesised that Polβ could promising target in ovarian cancers.

## Results

### Polβ expression links to aggressive epithelial ovarian cancers

**Fig. 1:**
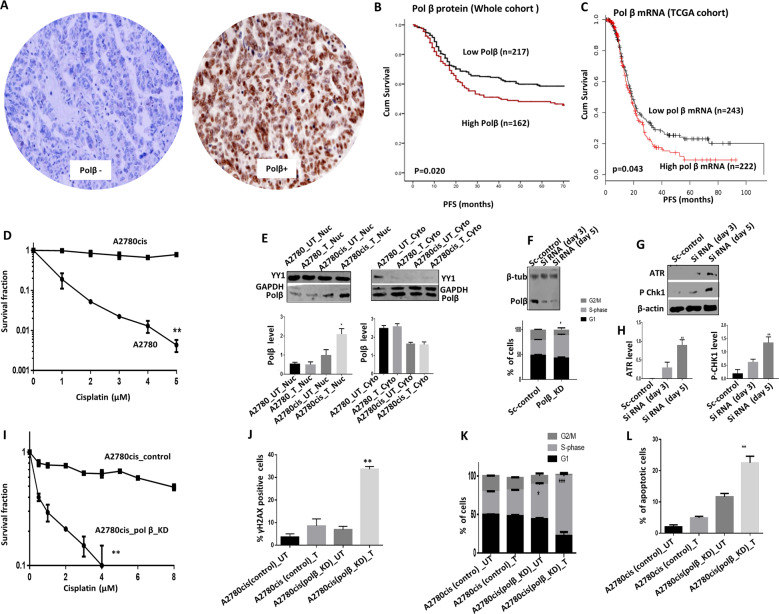
High Polβ expression is linked to aggressive ovarian cancers. **A** Immunohistochemical expression of Polβ in ovarian cancers. **B** Kaplan–Meier curve for overall survival in: whole cohort. **C** Kaplan–Meier curve for *Polβ* mRNA expression. **D** Cisplatin sensitivity by clonogenic survival assay in A2780 and A2780cis. **E** Polβ nuclear and cytoplasmic extracts in A2780 and A2780cis treated with 5 µM cisplatin. Lysates collected 48 h post treatment. **F** Polβ siRNA knock down in A2780cis cells. Cell cycle analysis for A2780cis control and Polβ**_**KD cells is shown here, **G** ATR and p-CHK1 protein expression in A2780cis Polβ**_**KD cells. **H** Quantification of ATR and p-CHK1 by western blot. **I** Cisplatin sensitivity by clonogenic survival assay in A2780cis cells control and A2780cis_Polβ_KD cells. **J** Quantification of γH2AX positive cells by flow cytometry in A2780cis cells control and Polβ_knock down treated with 5 µM cisplatin for 24 h. **K** Cell cycle analysis by flow cytometry in A2780cis cells control and Polβ_knockdown treated with 5 μM cisplatin. **L** AnnexinV analysis for apoptotic cells in A2780cis cells control and Polβ_knock down treated with 5 μM cisplatin. For Flow cytometry cells were seeded and transfected with scrambled control or Polβ siRNA. At day 3 controls and knockdown cells were re platted in six-well plates overnight and treated with 5 μM cisplatin and analyzed by flow cytometry on day 5. Transfection efficiency was confirmed by western blotting. Figures are representative of three or more independent experiments.

A total of 379 tumours were suitable for analysis of nuclear expression of polβ. 217/379 (57.3%) tumours were low for polβ and 132/379 (42.7%) of tumours were high in polβ expression (Fig. 1A). Low polβ expression was more common in mucinous cystadenocarcinoma, endometrioid and clear cell carcinomas (*p* = 0.001). Whereas, high polβ expression was seen in sub-optimally debulked (*p* = 0.023) (Supplementary Table 1). On the other hand, FIGO stage I disease were more common in tumours with low polβ expression (*p* = 0.003). Platinum resistance was more common in tumours with high polβ expression although it did not reach significance (*p* = 0.076). High polβ expression was associated with poor PFS (*p* = 0.020) (Fig. 1B) and poor overall survival (OS) (*p* = 0.029) (Supplementary Fig. S1A). At the transcriptional level, similarly, high *polβ* mRNA expression was associated with poor PFS and OS in both the test and validation cohort 1 (all *p* values < 0.05) (Supplementary Fig. S1). In the cancer genome atlas (TCGA) cohort (Validation cohort 2), high *polβ* mRNA expression was associated with poor PFS (Fig. 1C). Clinical data suggest that high polβ is a marker of adverse phenotype. We proceeded to pre-clinical studies.

### Polβ localises to the nucleus after platinum treatment in ovarian cancer cells

We chose A2780 (platinum sensitive) and A2780cis (platinum resistant) ovarian cancer cell lines for initial pre-clinical studies [12] (Fig. 1D). To evaluate for alterations in sub-cellular localisation of polβ upon cisplatin treatment, we generated nuclear and cytoplasmic extracts at baseline and following 48 h cisplatin therapy. We observed a significant accumulation of polβ protein in the nucleus in A2780cis cells compared A2780 cells (Fig. 1E) and (Supplementary Fig. S2A). There was no alteration in cytoplasmic level of polβ protein in A2780 or A2780cis cells (Fig. 1E) and (Supplementary Fig. S2A). For further validation, we monitored polβ sub-cellular localisation upon cisplatin treatment using immunofluorescence assay at 24 and 48 h. As shown in Supplementary Fig. S2B, C, we observed substantial nuclear accumulation of polβ after 48 h of cisplatin treatment in A2780cis cells compared to A2780 cells. The data suggest that alterations in sub-cellular localisation of polβ after platinum therapy may influence sensitivity.

### Bioinformatics analyses of the polβ interactome

As polβ mutants [13] have been reported previously to influence biochemical function and platinum sensitivity, we performed next generation exome sequencing and used the variant effect predictor tool [14] to identify genetic variants in platinum sensitive (A2780, PEO1) and platinum resistant (A2780cis, PEO4) cells (manuscript in preparation). We did not observe any polβ variants in A2780, A2780cis, PEO1 and PEO4 cells. While variants affecting polβ functionally associated genes were not statistically enriched in platinum-resistant cell lines, given the crucial role of polβ as a mediator of platinum resistance, we next examined loci encoding polβ-interacting proteins, as defined in the BioGRID database [15] (Supplementary Fig. S3A). Among these polβ interacting proteins, we identified coding variants affecting *LIG3, XRCC3, WRN;* synonymous variants affecting *EP300, UNG, XPC* and non-coding variants affecting the *APEX1, APTX, BCKDHA, BCKDHB, HUS1,* *KTN1, NEIL1, RAD9A, SRPK2, SRPK2, STUB1, TDP1, TLE1, TPP2, XPC, CRBN, TAF1D* loci in platinum resistant A2780cis and PEO4 cells compared to platinum sensitive A2780 and PEO1 cells (Supplemental Table 2). These include novel variants in *EP300, HUS1, KTN1, UNG, WRN, XRCC3* and *XPC*. Taken together, the data suggest that polβ and variants affecting the polβ functional interactome may contribute to platinum resistance either directly or indirectly through interactions with other factors involved in processing platinum induced DNA damage.

### Polβ depletion increases platinum sensitivity

**Fig. 2:**
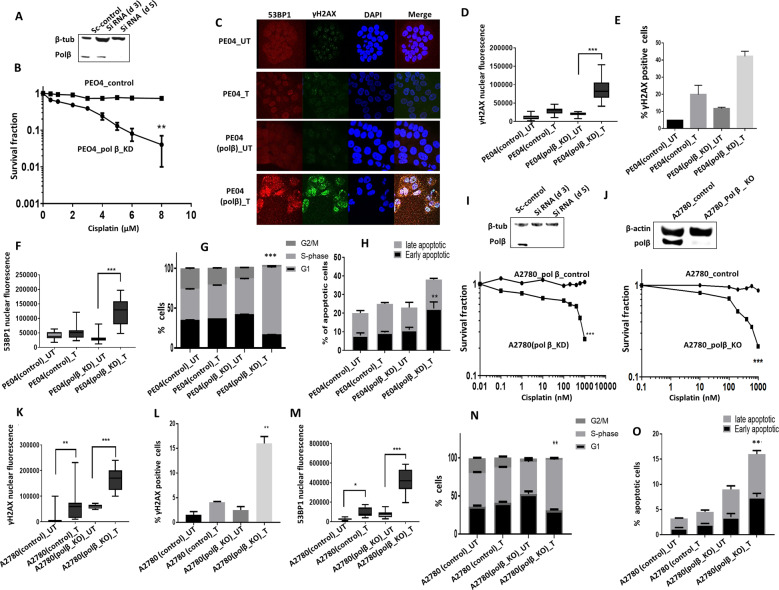
Polβ depletion in ovarian cancer cell lines. **A** Polβ siRNA transfection in PEO4 cells. **B** Cisplatin sensitivity by clonogenic survival assay in PEO4 scrambled control transfected cells and PEO4 Polβ_knockdown. PEO4 scrambled control and PEO4_Polβ_knockdown cells were treated with 5 μM cisplatin and on day 5 cells were analyzed by flow cytometry. **C** Representative photomicrographic images for immunofluorescence staining of γH2AX and 53BP1 in PEO4 scrambled control and polβ knockdown cells treated with 5 μM cisplatin. **D** Quantification of γH2AX nuclear fluorescence by ImageJ software. **E** Quantification of γH2AX positive cells by flow cytometry. **F** Quantification of 53BP1 nuclear fluorescence by ImageJ software. **G** Cell cycle analysis by flow cytometry. **H** Annexin V analysis by flow cytometry. **I** Polβ siRNA transfection in A2780 cells and Cisplatin sensitivity by clonogenic survival assay in A2780 scrambled control and Polβ_Knockdown cells. **J** Polβ knock out by CRISPR-Cas9 in A2780 cells and Cisplatin sensitivity by clonogenic survival assay in A2780 control and Polβ_Knock out cells. **K** Quantification of γH2AX nuclear fluorescence by ImageJ software. A2780 control and A2780 Polβ_knock out cells were treated with 1 μM cisplatin and analyzed by Immunofluorescence or flow cytometry on day 5. **L** Quantification of γH2AX positive cells by flow cytometry. **M** Quantification of 53BP1 nuclear fluorescence by ImageJ software. **N** Cell cycle analysis by flow cytometry. **O** Annexin V analysis by flow cytometry. A2780 control and Polβ_KO cells were plated overnight and treated with 1 μM cisplatin for 24 h. After incubation, cells were collected and stained as per the flow cytometry protocol detailed in the methods. Figures are representative of three or more independent experiments.

BER may operate predominantly during G1 phase of the cell cycle [10]. Moreover, a BER complex (consisting of polβ, APE1, UNG2 and XRCC1) was shown to physically associate with MCM7 suggesting that BER may also operate at sites of base damage and single-strand breaks occurring at replication forks [16]. In addition, polβ activity may also be prominent during the S-phase of the cell cycle [17]. We therefore generated transient knockdowns (KD) of polβ in A2780cis cells using siRNAs (Fig. 1F, Supplementary Fig. S4A) and evaluated cell cycle progression in control and polβ _KD cells. Compared to control cells, we observed significant S-phase arrest in polβ_KD cells (Fig. 1F) which was associated with accumulation of ATR and pChk1^ser 345^ (Fig. 1G, H) and (Supplementary Fig. S4B, C), a feature that would be consistent with replication stress in A2780cis polβ_KD cells. In clonogenic assays, Polβ_KD_A2780cis cells (transient KD generated using siRNA) were strikingly sensitive to platinum therapy (Fig. 1I). We also confirmed this observation using another siRNA construct which also showed robust Polβ_KD and also lead to platinum sensitisation (Supplementary Fig. S4D, E). Polβ_KD was associated with double-strand break (DSB) accumulation (Fig. 1J), S-phase cell cycle arrest (Fig. 1K) and apoptosis (Fig. 1L). We then tested in another platinum resistant PEO4 ovarian cancer cells. As expected, polβ depletion (Fig. 2A) and (Supplementary Fig. S4F) increased platinum sensitivity (Fig. 2B) was associated with increased γH2AX nuclear foci (Figs. 2C–E), 53BP1 foci accumulation (Fig. 2F) S-Phase arrest (Fig. 2G) and apoptotic cells (Fig. 2H). In platinum sensitive A2780 cells treated with very low doses of cisplatin (nanomolar range), again polβ depletion by SiRNA (Fig. 2I) and (Supplementary Fig. 4G) or with CRISPR-cas9 (Fig. 2J) resulted in increased platinum sensitivity. We confirmed this result using two siRNA constructs which showed robust polβ knock down and similarly lead to platinum sensitisation (Supplementary Fig. S4H, I). Polβ depletion increased γH2AX nuclear foci accumulation (Fig. 2K, L) and (Supplementary Fig. S5A), 53BP1 nuclear foci accumulation (Fig. 2M), S-phase arrest (Fig. 2N) and apoptotic cells (Fig. 2O).

We have tested another Isogeneic ovarian cancer cell line model. PEO1, which is BRCA2 deficient, is a platinum sensitive ovarian cancer cell line. We have recently generated a platinum resistant and PARP inhibitor (Olaparib) resistant cell line (PEO1R) from PEO1 cells. PEO1R was generated by chronic exposure to an inhibitor of MRE11 (mirin) over 6 months (Alblihy et al. manuscript submitted). As shown in Supplementary Fig. S5B, PEO1R is resistant to cisplatin treatment compared to PEO1 (Supplementary Fig. S5B). Interestingly, Polβ knockdown in PEO1R cells significantly increases sensitivity to cisplatin (Supplementary Fig. S5B).

**Fig. 3:**
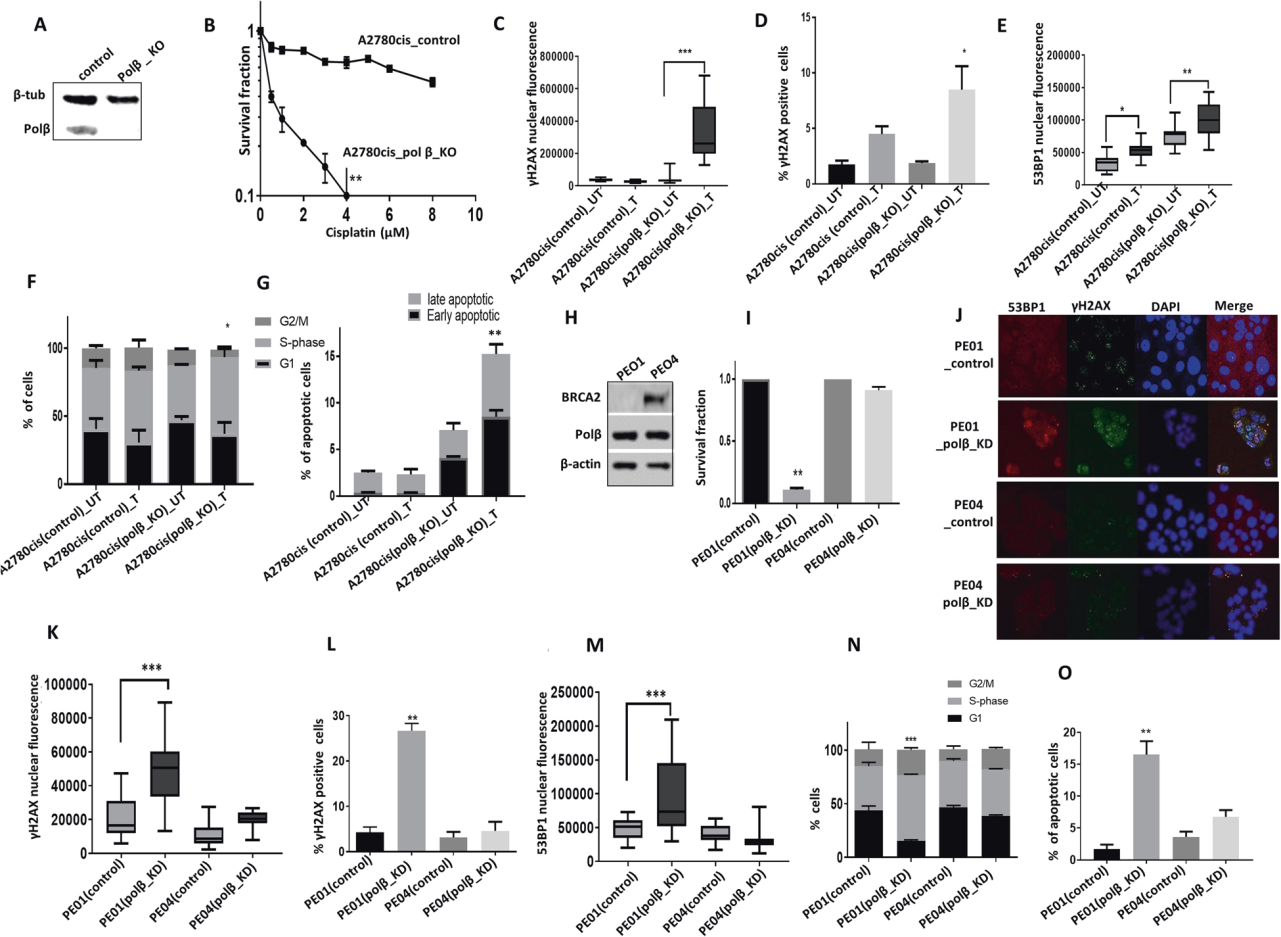
Polβ knock out increases platinum sensitivity. **A** Polβ CRISPR_knockout in A2780cis cells. **B** Cisplatin sensitivity in A2780cis control and Polβ-knockout cells. **C** Quantification of γH2AX nuclear fluorescence by ImageJ software. **D** Quantification of γH2AX positive cells by flow cytometry. **E** Quantification of 53BP1 nuclear fluorescence by imageJ software. **F** Cell cycle analysis by flow cytometry. **G** Annexin V analysis by flow cytometry. For A2780cis control and Polβ_knockout cells, Cells were treated with 5 μM cisplatin for 24 h and analyzed by flow cytometry or cells were plated on coverslips overnight and treated with 5 μM cisplatin for 24 h. Cells were then fixed and stained for immunofluorescence as detailed in the methods. All figures are representative of three or more experiments. **H** BRCA2 and polβ expression by western blot in PEO1 and PEO4 cells. **I** Survival fraction in PEO1 and PEO4 control and Polβ_knockdown cells. **J** Representative photomicrographic images of PEO1 and PEO4 control and Polβ_knockdown cells. **K** Quantification of γH2AX nuclear fluorescence by ImageJ software. **L** γH2AX positive cells analysis by flow cytometry. **M** Quantification of 53BP1 nuclear fluorescence by ImageJ software. **N** Cell cycle analysis by flow cytometry. **O** Annexin V analysis by flow cytometry. PEO1 and PEO4 control and Polβ_knockdown cells were transfected with Polβ SiRNA; on day 4 cells were plated in six-well plates overnight and analyzed by flow cytometry on day 5. Figures are representative of three or more experiments.

We then generated A2780cis polβ-knockout (KO) cells using CRISPR-Cas-9 methodology (Fig. 3A) and (Supplementary Fig. S5C). As shown in Fig. 3B, polβ_KO strikingly increased sensitivity to platinum which was associated increased nuclear γH2AX nuclear foci accumulation (Fig. 3C, D) and (Supplementary Fig. S5D), 53BP1 foci accumulation (Fig. 3E), S-phase arrest (Fig. 3F) and accumulation of apoptotic cells (Fig. 3G). As platinum treatment can induce the generation of free-radicals in cells and cause oxidative DNA base damage, we also explored if the sensitivity to cisplatin could be altered upon antioxidant treatment. To address this hypothesis, we pre-treated cells with curcumin (antioxidant) for 24 h followed by cisplatin treatment. Polβ deficient cells remained sensitive to platinum therapy (Supplementary Fig. S6A). We then tested another DNA cross-linker Mitomycin C. We again observed that polβ KO cells were also sensitive to mitomycin C treatment (Supplementary Fig. S6B). Together, the data provide evidence that polβ is a predictor of platinum sensitivity.

### A role for Polβ in the regulation of invasion, migration and EMT

There is also emerging evidence for the role of epithelial to mesenchymal transition (EMT) in ovarian cancer chemotherapy resistance [18]. To evaluate if polβ can also influence the aggressive behaviour of ovarian cancer we proceeded to investigate invasion, migration and EMT in control and polβ_KO ovarian cancer cell lines. Polβ_KO in A2780 and in A2780cis cells significantly reduced invasion (Supplementary Fig. S7A). Given the well-defined roles of E-cadherin [19], N-cadherin [20], TGFβ [21] and MMP-9 [22] in invasion and EMT regulation, we evaluated if polβ could influence the expression of these key EMT markers in ovarian cancer cells. As shown in Supplementary Fig. S7B–D there was a clear induction of E-cadherin expression in polβ_KO A2780 and A2780cis cells compared to control. On the other hand, N-cadherin and MMP-9 were significantly reduced in A2780 polβ_KO cells (Supplementary Fig. S7B–D). TGFβ was significantly reduced in A2780cis polβ_KO cells. Supplementary Fig. S7B–D. The data provide evidence that polβ may have a role in EMT. To provide additional evidence we performed real-time PCR using RT^2^ Profiler EMT PCR Array for 86 EMT genes. Full list of EMT genes included in the PCR array is summarised in Supplementary Table S3. Compared to control cells, downregulation of several genes with defined roles in EMT was evident in polβ_KO cells (Supplementary Fig. S8A, B). Interestingly, we also observed upregulation of some genes with roles in EMT (Supplementary Fig. S8A, B). Together the data would imply a complex role for polβ is influencing the expression of genes involved in EMT.

### Polβ blockade is synthetically lethal in BRCA2 deficient cells

**Fig. 4:**
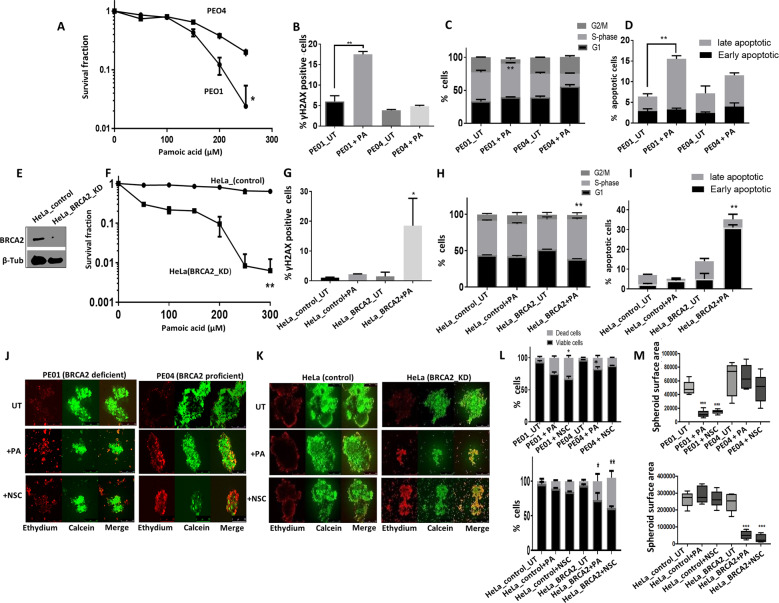
Polβ inhibitors induce synthetic lethality in BRCA2 deficient ovarian cancer cells. **A** Pamoic acid sensitivity by clonogenic survival assay in PEO1 and PEO4 cells. **B** γH2AX analysis by flow cytometry for PEO1 and PEO4 cells treated with Pamoic acid (250â€‰μM). **C** Cell cycle analysis by flow cytometry in PEO1 and PEO4 cells treated with Pamoic acid (250â€‰μM) for 24â€‰h. **D** Annexiγn V analysis by flow cytometry in PEO1 and PEO4 cells treated with Pamoic acid (250â€‰μM) for 24â€‰h. **E** BRCA2 western blot in HeLa control and HeLa BRCA2_knockdown cells. **F** Pamoic acid sensitivity in HeLa control and HeLa BRCA2_knock down cells by clonogenic survival assay is shown here. γH2AX analysis by flow cytometry (**G**), cell cycle analysis by flow cytometry (**H**) and annexinV analysis by flow cytometry (**I**) in HeLa control and HeLa BRCA2_knockdown cells treated with Pamoic acid (250â€‰μM) for 24â€‰h. Representative photomicrographic images of Pamoic acid (250â€‰μM) and NSC666719 (250â€‰μM) treated 3D-spheres of: PEO1 and PEO4 cells (**J**), HeLa control and HeLa (BRCA2_KD) (**K**). **L** Quantification of spheroids cell viability by flow cytometry. **M** Quantification of spheroids size by ImageJ. Figures are representative of three or more experiments. Error bars represent standard deviation between experiments.

BRCA2 is a key player in homologous recombination (HR). Patients with germ-line mutations in *BRCA2* are predisposed to ovarian cancer development with a cumulative life time risk of about 20–30% [23]. PARP inhibitor maintenance therapy improves PFS in BRCA2 germ-line deficient ovarian cancers [1–3]. However, not all patients respond either due to intrinsic resistance or acquired resistance to PARP inhibitors [4]. We hypothesised that polβ could be a promising alternative synthetic lethality target in BRCA2 deficient ovarian cancers. Polβ deficiency impairs BER and leads to accumulation of single-strand breaks, which if unrepaired, result in generation of DSBs during replication. In cells deficient in HR repair (HRR), DSBs would persist and lead to synthetic lethality. We therefore tested synthetic lethality in BRCA2-deficient (PEO1) and BRCA2-proficient (PEO4) ovarian cancer cells. PEO1 and PEO4 cells have robust Polβ expression (Fig. 3H) and (Supplementary Fig. S9A). Cell viability, as investigated by clonogenic assay, was significantly impaired when polβ was depleted in PEO1 cells, but not in PEO4 cells (Fig. 3I). Polβ depletion in PEO1 cells resulted in increased γH2AX foci accumulation (Fig. 3J–L) 53BP1 foci accumulation (Fig. 3M), S-phase cell cycle arrest (Fig. 3N) and induction of apoptosis (Fig. 3O). We then tested cytotoxicity of polβ inhibitors (polβi) in BRCA2-proficient and deficient cell lines. Pamoic acid is a well described polβi [24]. The cytotoxicity of Pamoic acid was first tested in control and polβ_KO cells. We did not observe any significant cytotoxicity in polβ_KO cells compared to control cells (Supplementary Fig. S9B). The data suggest that Pamoic acid may have specific activity against polβ. Compared to PEO4 cells, PEO1 cells were sensitive to Pamoic acid treatment (Fig. 4A) which was associated with DSB accumulation (Fig. 4B), S-phase arrest (Fig. 4C) and apoptosis (Fig. 4D). We then validated Pamoic acid activity in BRCA2 deficient and control HeLa cells (Fig. 4E). As shown in Fig. 4F, Pamoic acid was selectively toxic in HeLa BRCA2-deficient cells compared to control HeLa cells. Increased sensitivity was associated with DSB accumulation (Fig. 4G), S-phase arrest (Fig. 4H) and increased apoptotic cells (Fig. 4I).

To recapitulate an in vivo system, we then generated 3D-spheroids of PEO1, PEO4, HeLa control and HeLa BRCA2_KD cells (Fig. 4J, K). BRCA2-proficient cells (PEO4 and HeLa controls) and BRCA2-deficient cells (PEO1 and HeLa BRCA2_KD cells) retain spheroid forming capacity (Fig. 4J, K). However, upon Pamoic acid treatment, in BRCA2-deficient spheroids, there was an accumulation of apoptotic cells (Fig. 4L) as well as a reduction in spheroid size (Fig. 4M) compared to BRCA2-proficient spheroids (Fig. 4L, M). We then validated using NSC666719 [4-chloro-5-methyl-N-[5-(naphthalen-2-ylamino)-1H1,2,4-triazol-3-yl]-2 sulfanylbenzenesulfonamide], another specific polβi [25–27]. In BRCA2 deficient spheroids, NSC666719 treatment reduced spheroid size and viability (Fig. 4J–M). NSC666719 treatment also resulted in DSB accumulation (Supplementary Fig. S9C), G2/M cell cycle arrest (Supplementary Fig. S9D) and apoptotic cells (Supplementary Fig. S9E) in BRCA2-deficient cells compared to BRCA2-proficient cells.

### PARG inhibitor (PARGi) is selectively toxic in polβ deficient cells

**Fig. 5:**
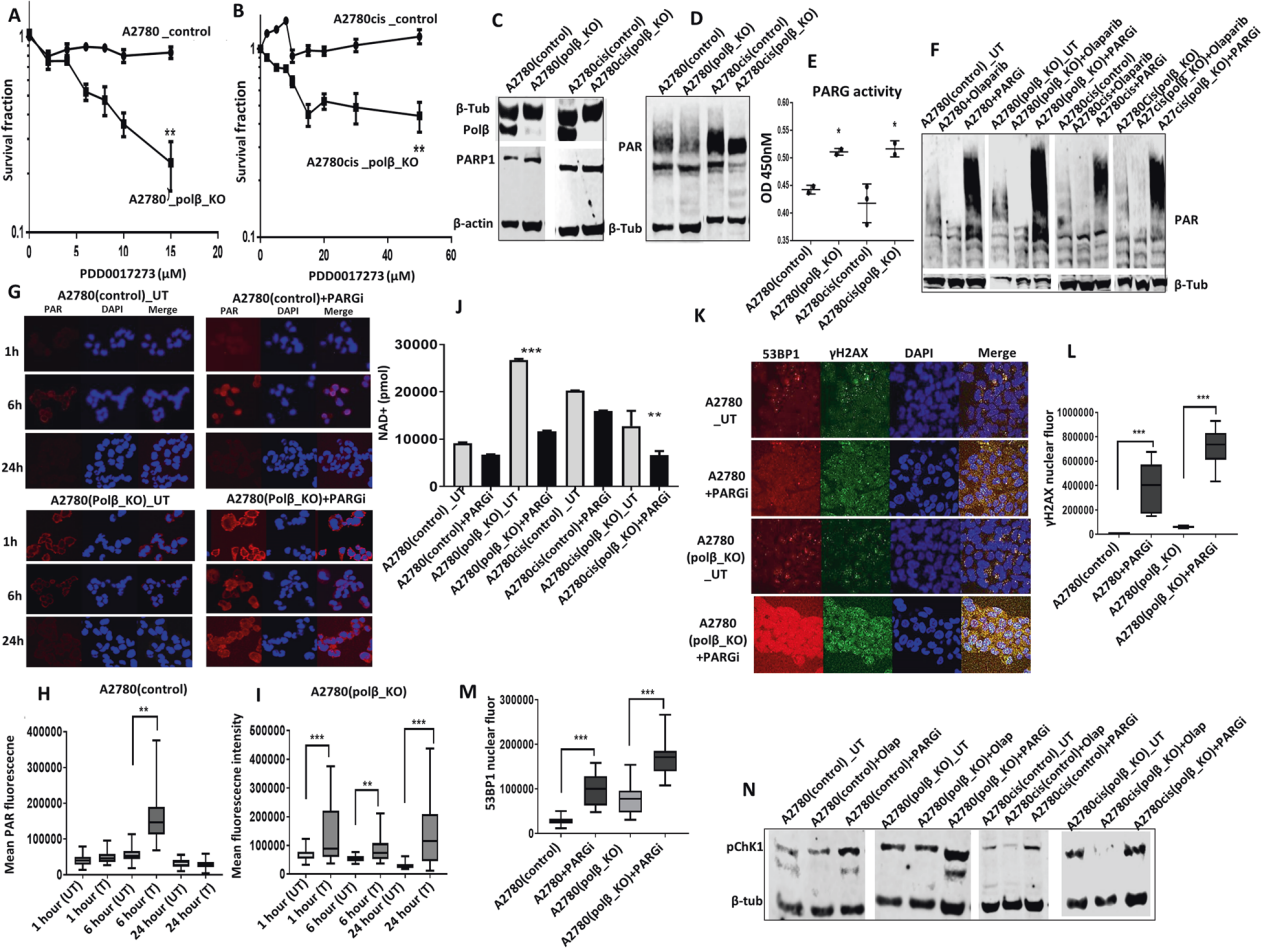
Polβ knock out cells is sensitive to PARG inhibitor. **A** PARG inhibitor sensitivity by clonogenic survival assay in A2780 control and Polβ_knock out cells. **B** PARG inhibitor sensitivity by clonogenic survival assay in A2780cis control and Polβ_knock out cells. **C** Polβ, PARP1 levels by western blot in A2780 and A2780cis control and Polβ_knockout cells. **D** Poly (ADP) ribose polymers levels in A2780 and A2780cis control and Polβ_knock out cells. **E** PARG ELISA assay in A2780 control, A2780 (Polβ_KO), A2780cis control and A2780cis (Polβ_KO) cells. **F** Poly (ADP) ribose polymers levels in A2780 and A2780cis control and Polβ_knock out cells treated with Olaparib or PARG inhibitor. Cells were treated with Olaparib (10 μM) or PARGi (20 μM) for 16 h then extracted for western blot. **G** Immunofluorescence staining for poly (ADP) ribose polymers in A2780 control and Polβ_knockout cells untreated or treated with PARGi (20 μM) for 1, 6, and 24 h. **H**, **I** Quantification of Poly (ADP) ribose polymers fluorescence by imageJ software. **J** Quantification of NAD+ levels in A2780 and A2780cis control and Polβ_knock out cells. **K** Representative photomicrographic images of 53BP1 and γH2AX immunofluorescence in A2780 and A2780cis control and Polβ_knock out cells treated with PARGi (20 μM) for 16 h. **L** Quantification of γH2AX nuclear fluorescence by ImageJ software. **M** Quantification of 53BP1 nuclear fluorescence by ImageJ software. **N** p-CHK1 levels by western blot in A2780 and A2780cis control and Polβ_knock out treated with Olaparib (10 μM) or PARGi (20 μM) for 16 h. Figures are representative of three or more experiments. Error bars represent standard deviation between experiments.

At sites of DNA damage, PARP1 is recruited where it induces the synthesis of poly(ADP-ribose) (PAR). PARylation of PARP1 and other DNA repair factors is essential for coordination of DNA repair [28]. PARylation is transient and reversible process. PAR glycohydrolase (PARG) is a key factor in the PAR degradation pathway [29–31] A coordinated activity of PARP and PARG is also essential for efficient DNA repair. Recently, PARG has also emerged as a promising drug target in cancer [32, 33]. Importantly, a recent study by Pillai et al. demonstrated that ovarian cancer cells respond differently to PARGi (PDD00017273) compared to PARPi (Olaparib) [34]. DNA replication vulnerabilities was shown to particularly render ovarian cancer cells sensitive to PDD00017273 treatment leading to persistent replication fork stalling and replication catastrophe in that study [34]. As polβ-deficient ovarian cancer cells are not only sensitive to platinum treatment but also show features of replication stress as evidenced by accumulation of ATR and pChk1^ser 345^, we tested a synthetic lethality application for either PARPi or PARGi. We first evaluated cellular activity of Olaparib and PDD00017273 in polβ-deficient and -proficient ovarian cancer cells. Olaparib did not induce selective cytotoxicity in polβ_KO or control cells (Supplementary Fig. S9F, G). However, as shown in Fig. 5A, B, PDD00017273 treatment was selectively toxic in polβ_KO A2780 or polβ_KO A2780cis cells compared to controls. To also validate this observation in PEO4 ovarian cancer cell line, we generated transient knockdown of polβ and again observed increased sensitivity to PDD00017273 treatment in PEO4 cells (Supplementary Fig. S9H). We proceeded to functional studies to understand potential mechanisms of PDD00017273 toxicity.

### Mechanistic studies of PARGi in polβ-deficient cells

**Fig. 6:**
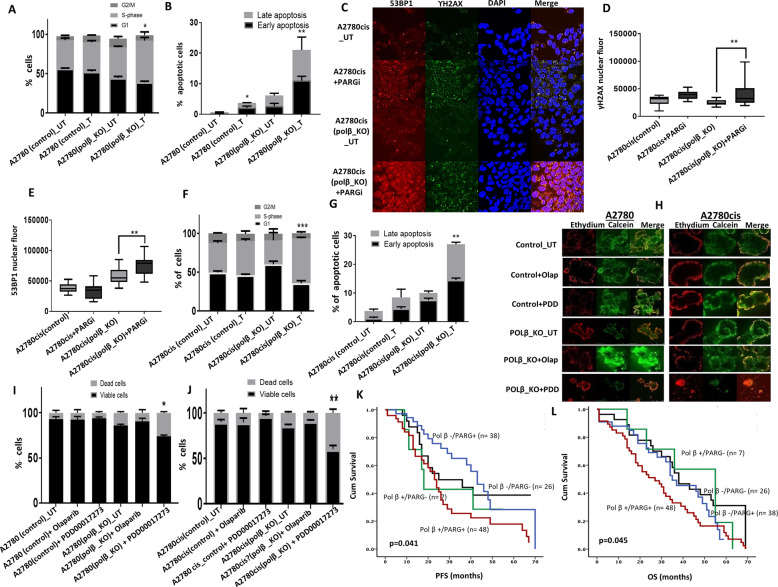
Mechanisitc studies of PARG inhibitor in ovarian cancer. **A** Cell cycle analysis by flow cytometry. **B** Annexin V analysis by flow cytometry for A2780 cells control and Polβ_knockout cells treated with 20 μM PARGi for 24 h. **C** Representative photomicrographic images for 53BP1 and γH2AX immunofluorescence staining in A2780 control and Polβ_knockout cells treated with 20 μM PARGi. **D** Quantification by γH2AX nuclear fluorescence by ImageJ software. **E** Quantification of 53BP1 nuclear fluorescence by ImageJ software. **F** Cell cycle analysis by flow cytometry. **G** Annexin V analysis by flow cytometry. A2780cis cells control and Polβ_knockout cells were treated with 20 μM PARGi for 24 h cells were collected and analysed as per the flow cytometry protocol or cells were seeded on coverslips and treated with PARGi(20 μM) for 24 h before staining for immunofluorescence protocol. **H** Representative photomicrographic images for A2780 control and Polβ_knockout spheroids treated with Olaparib (10 μM) or PARGi (20 μM). **I**, **J** Quantification of spheroids cell viability is shown here. Figures are representative of three or more independent experiments. **K** Polβ and PARG co-expression and Kaplan–Meier curve for ovarian cancer progression-free survival (PFS). **L** Polβ and PARG co-expression and Kaplan–Meier curve for overall survival in ovarian cancer.

At baseline, there were no changes in PARP1 levels in both control and polβ_KO cells (Fig. 5C, Supplementary Figs. S9I, S10A). However, in polβ_KO cells there was a reduction in PAR level compared to control cells (Fig. 5D) and (Supplementary Fig. S11B, C). Using PARG ELISA assay we show that polβ_KO cells have increased PARG activity compared to control cells (Fig. 5E). We then evaluated PAR level in polβ_KO and controls treated with either Olaparib or PDD00017273 (Fig. 5F) and (Supplementary Fig. S10D, E). In Olaparib-treated cells, as expected, there was a reduction in PAR levels in both control and polβ_KO cells which also confirmed in a PAR ELISA assay (Supplementary Fig. S10G). PDD00017273 treatment increased PAR level in control and polβ_KO cells (Fig. 5F) and (Supplementary Fig. S10D–F). To further validate, we monitored PAR level by confocal microscopy at 1, 6, and 24 h following PDD00017273 treatment in A2780_ polβ_KO and control cells (Fig. 5G–I). In A2780 control cells, following PDD00017273 treatment, there was transient increase in PAR accumulation at 6 h which returned to baseline levels at 24 h compared to untreated cells. However, in A2780_ polβ_KO cells, there was accumulation of PAR at 24 h compared to untreated cells (Fig. 5G–I). PARP1 catalyses the polymerisation of ADP-ribose units (PAR) from NAD+. On the other hand, PARG catalyses the hydrolysis of PAR producing free mono- and oligo(ADP-ribose) [28]. We evaluated NAD+ levels in untreated as well as PDD00017273-treated control and polβ_KO cells (Fig. 5J). In untreated A2780_ polβ_KO cells, there was a significant increase in NAD+ levels compared to control cells. Upon PDD00017273 treatment there was a significant reduction in NAD+ levels in A2780_ polβ_KO cells compared to controls. Interestingly in untreated A2780cis_ polβ_KO cells, there was no increase in NAD+ levels compared to controls. However following PDD00017273 treatment NAD+ levels significantly reduced compared to controls (Fig. 5J). Together, the data suggest a complex cell line dependent network operating between polβ, PAR and NAD+. Previous studies have shown that PAR accumulation can not only impair efficient DNA repair but can also be directly toxic to cells [35, 36]. Depletion of NAD+ is known to alter NAD+/SIRT1 axis which also leads to impaired DNA repair and cell survival [37, 38]. PDD00017273 treatment in A2780_polβ_KO cells promoted γH2AX nuclear foci accumulation (Fig. 5K, L) and (Supplementary Fig. S11G), an indirect biomarker for DSBs and replication stress-induced defects. As a further validation of DSBs, we also observed 53BP1 nuclear foci accumulation in PDD00017273-treated A2780_polβ_KO cells (Fig. 5K, M). Consistent with PDD00017273 induced replication arrest, CHK1 was phosphorylated on serine 345 in PARGi-treated polβ deficient cells (Fig. 5N, Supplementary Fig. S10H), which was then associated with S-phase arrest (Fig. 6A) and induction of apoptosis (Fig. 6B). Similarly, in A2780cis_ polβ_KO cells compared to controls, PDD00017273 treatment was associated with γH2AX nuclear foci accumulation (Fig. 6C, D), 53BP1 nuclear foci accumulation (Fig. 6E), increased phosphorylated CHK1 (Fig. 5M, Supplementary Fig. S10H), S-phase arrest (Fig. 6F) and apoptosis (Fig. 6G). In 3-D spheroid models, PDD00017273 treatment reduced spheroid size and promoted cell death in polβ_KO spheroids compared to control spheroids (Fig. 6H–J). Olaparib did not influence cell viability in 3-D spheroid assays (Fig. 6H–J). Together the data suggest that PARGi in polβ deficient induces selective cytotoxicity through replication stress, DSB accumulation and cell death. Talazoparib, has at least 100 times more potency to trap PARP at replication forks compared to Olaparib [28]. As expected, A2780_polβ_KO and A2780cis_ polβ_KO cells were sensitive to Talazoparib therapy compared to controls (Supplementary Fig. S11A, B). The data would suggest that replication stress induction contributes to the observed cytotoxicity.

### Clinicopathological significance of PARG expression in human ovarian cancers

PARG expression was cytoplasmic. Low PARG expression was seen in 60/274 (22%) tumours and high PARG expression was observed in 214/274 (72%) of tumours. High PARG expression was associated with serous cystadenocarcinomas (*p* = 0.011) (Supplementary Table S4). PARG expression did not influence PFS (Supplementary Fig. S12A). However, high PARG expression was associated with poor OS in the whole cohort (*p* = 0.032) (Supplementary Fig. S12B). The data suggest that PARG may have prognostic significance in ovarian cancers. We then performed polβ/PARG co-expression analysis. Polβ/PARG co-expression was associated with serous cystadenocarcinomas (*p* = 0.017) and higher stage (*p* = 0.004) (Supplementary Table S5). Tumours with high polβ/PARG co-expression have poor PFS (Fig. 6K) (*p* = 0.041) as well as OS (Fig. 6L) (*p* = 0.045) compared to tumours with low polβ/PARG co-expression. In multivariate analyses (Supplementary Table S6), polβ (*p* = 0.012) and platinum sensitivity (0.0001) independently influenced PFS. Platinum sensitivity remained independently linked to OS (*p* = 0.0001) (Supplementary Table S6). PARG expression was borderline non-significant in multivariate analysis (*p* = 0.051 and 0.056 respectively) (Supplementary Table S6).

## Discussion

Polβ, a member of the X-family of DNA polymerases is a key player in BER [10]. Pre-clinically we demonstrated that polβ depletion increased platinum sensitivity in platinum resistant A2780cis, PEO4 and PEO1R ovarian cancer cells. In platinum sensitive A2780 cells, although there were some variations due to different experimental conditions, Polβ depletion using multiple siRNA constructs consistently increased platinum sensitivity. Interestingly, in cisplatin untreated A2780cis cells polβ deficiency caused S-phase arrest and a replication arrest phenotype. However, we did not observe this phenomenon in A2780 or PEO4 cells, implying a cell line dependent role for polβ during replication in ovarian cancer cells.

Higher levels of polβ have been described in colonic and prostate compared with normal tissue [39, 40]. Mutations in polβ may influence aggressive solid tumour phenotype and response to chemotherapy [13]. In ovarian cancers, mutations in polβ have been recognised [41] although clinical significance remains unclear [41]. In clinical cohorts of ovarian cancers, we show that low Polβ expression is associated with better PFS. In the current study, we did not observe any cytoplasmic staining for polβ protein in ovarian tumours. For nuclear expression, we have observed some peri-nuclear or annular staining for polβ. Whether this represents altered polβ expression or variable polβ expression due to DNA damage is unknown but will be an interesting area for further exploration in the future. Importantly, we provide evidence that low polβ expression at the protein as well as at the mRNA levels is linked with better PFS.

Polβ deficiency in mice is embryonically lethal [42] and embryonic fibroblasts derived from such mice are hypersensitive to alkylating agents [43]. Depletion of polβ delayed the repair of oxaliplatin-induced DNA damage and increased sensitivity in colorectal cancer cell lines [44]. On the other hand, polβ overexpression increased resistance to DNA-damaging agents [45]. Our data concur with a previous study in colorectal cell lines showing platinum sensitivity with polβ depletion [46].

Chronic platinum exposure can alter function of microtubules and microfilaments and can reduce migration [47, 48], a feature also seen in A2780cis cells. We show that wild-type cells have expression of N-Cadherin, TGFβ. and MMP9. Polβ depletion reduced N-Cadherin, TGFβ, and MMP9 levels along with induction of E-cadherin. Cadherin switch is the hallmark of EMT [49]. TGFβ and MMP-9 also have a role migration/invasion [49]. Whilst downregulation of several EMT genes was also evident in polβ-depleted cells, we speculate an indirect role for polβ in EMT which require future mechanistic study confirmation. Interestingly, previous studies show PARP1 depletion [50] and Histone H2AX depletion [51] in promoting EMT through transcriptional regulation suggesting a complex role for DNA repair in EMT.

In the current study we not only observed increased nuclear localisation of polβ following cisplatin treatment but basal levels of polβ protein also appears to be higher in platinum resistant cells. Although we did not identify any activating mutations of *polβ*, we speculate that post transcriptional or translational mechanisms could result in overexpression of polβ. Another interesting observation was that despite the lack of *polβ* variants, bioinformatics analyses revealed several key polβ-interacting proteins harboured novel non-synonymous variants, affecting the *LIG3, WRN* and *XRCC3* loci. However, a limitation to the current study is that further detailed functional and mechanistic studies would be required to fully evaluate if polβ functional interactome may contribute to platinum resistance in ovarian cancer cells.

We show that polβi can induce synthetic lethality in BRCA2-deficient cells. Although promising, future in vivo xenograft studies are required to confirm our findings. PARG blockade is also selectively toxic in polβ-deficient cells. Polβ depletion or inhibition increase SSBs. PARG is essential for efficiency of SSB repair [52]. In polβ deficient cells we observed increased PARG activity. PARG inhibition or depletion can also increase reversed replication forks and post-replicative single-strand breaks [32, 33]. In polβ deficient cells with impaired BER and increased SSB, PARG inhibition leads to profound accumulation of SSBs which get converted to DSB leading to synthetic lethality. Accordingly, we observed DSB accumulation, S-phase arrest and apoptosis in PDP00017273-treated polβ-deficient cells compared to -proficient cells. Additionally, PARG inhibition was associated with accumulation of PAR polymers in polβ-deficient cells. Accumulation of PAR was accompanied by NAD+ depletion via two potential mechanisms; (1) PARP uses NAD+ as ADP-ribose donor and catalyse PARylation of target proteins including itself. 2) PARG mediates rapid turnover of PAR to mono-ADP-ribose units which is recycled as the ATP precursor, an important substrate to generate NAD+. PARGi will therefore increase NAD+ [31]. PARG mediated reversal of auto-modification of DNA bound PARP1 leads to poly-ubiquitination of PARP1 by the E3 ligase CHFR. Removal and degradation of PARP1 contribute further to the restoration of NAD+ levels. In PARGi-treated cells, therefore, reduced level of NAD+ may persists [53]. There are at least two mechanisms for NAD+ depletion induced cell death; (1) caspase-independent programmed necrosis, where excess PAR activates apoptosis-inducing factor, which triggers the apoptotic cascade [35, 36]. (2) NAD+ depletion can alter Sirtuin level and impair BER [37, 38] contributing further to SSB accumulation. Moreover, PAR accumulation is also known to be toxic to cells and can also impair DNA repair [35]. However, it should be noted that persistent accumulation of PAR polymers in A2780 Polβ_KO cells but transient formation of PAR polymers in the control A2780 was observed in the current study. The exact molecular mechanisms of this phenomenon are not clear although an increased level of PAR formation is expected with inhibition of PARG (which hydrolyses PAR polymers). As described, PAR accumulation and NAD depletion can contribute directly and indirectly to the impairment of DNA machinery, which could be more lethal in the context of polβ deficiency [54, 55]. A recent study proposed that loss of PARG activity is a possible mechanism for PARPi resistance in BRCA2 deficient tumours. PARG operates in the same direction as PARPi by preventing PAR accumulation. Hence, decreased PARG activity can allow tumour cells to escape the PARPi mediated synthetic lethality [29]. These findings concur with our observations in polβ deficient ovarian cancer cells. Another study by Pillay et al. has revealed that ovarian cancer cells respond differently to PARGi (PDD00017273) compared to PARP inhibitor (Olaparib) and may be dependent on DNA replication vulnerabilities in cells [34]. Therefore endogenous PARG levels could predict Olaparib response in HR deficient as well as in polβ deficient ovarian cancer cells. PARGi is also synthetically lethal in BRCA1 [32], BRCA2, PALB2, FAM175A and BARD1 deficient breast cancer cells [33].

In conclusion, polβ blockade is a novel approach warrants for development in ovarian cancers.

## Materials and methods

Full details are available in Supplementary material and methods.

### Clinical study

Polβ immunohistochemistry was completed in 525 epithelial ovarian cancers. *Polβ* mRNA expression in human epithelial ovarian was investigated in three ovarian tumour gene expression data sets [test set, validation cohort 1 and validation cohort 2 (TCGA). See Supplementary methods for full details. Ethical approval which was obtained from the Nottingham Research Ethics Committee (REC Approval Number 06/Q240/153). All patients provided informed consent.

### Pre-clinical study

A2780, A2780cis, PEO1 and PEO4 were purchased from American Type Culture Collection (ATCC, Manassas, USA). BRCA2-deficient HeLa SilenciX cells and controls BRCA2-proficient HeLa SilenciX cells were purchased from Tebu-Bio (www.tebu-bio.com).

Methodology for transient knockdown of Polβ and generation of Polβ knockouts are described in Supplementary methods.

Compounds, reagents, clonogenic assays, cell proliferation assays, confocal microscopy, functional assays (FACS, cell cycle progression, apoptosis assays. ELISA), invasion assay, migration assay, 3D-spheroid assays, next generation sequencing and bioinformatics are described in Supplementary methods.

## Supplementary information

Supplementary Methods

Supplementary Tables

Supplementary Figure legends

Supplementary Figure S1

Supplementary Figure S2

Supplementary Figure S3

Supplementary Figure S4

Supplementary Figure S5

Supplementary Figure S6

Supplementary Figure S7

Supplementary Figure S8

Supplementary Figure S9

Supplementary Figure S10

Supplementary Figure S11

Supplementary Figure S12
