# Supplementary material for: Molecular disruption of DNA polymerase β for platinum sensitisation and synthetic lethality in epithelial ovarian cancers
Source: Oncogene. 2021 Mar 5;40(14):2496–508. doi: 10.1038/s41388-021-01710-y (PMC8032555; doi:10.1038/s41388-021-01710-y)
Supplement: Supplementary file 1 — Supplementary Methods [file 41388_2021_1710_MOESM1_ESM.docx]

**Supplementary materials& methods**

**Patients:** Investigation of the expression of polβ protein in ovarian epithelial cancer was carried out on tissue microarrays of 525 consecutive ovarian epithelial cancer cases treated at Nottingham University Hospitals (NUH) between 1997 and 2010. Patients were comprehensively staged as per the International Federation of Obstetricians and Gynaecologists (FIGO) Staging System for Ovarian Cancer. Overall Survival was calculated from the operation date until the 1st of October 2016 when any remaining survivors were censored. Platinum resistance was defined as patients who had progression during first-line platinum chemotherapy or relapse within 6 months after completion of chemotherapy. Progression-free survival was calculated from the date of the initial surgery to disease progression or from the date of the initial surgery to the last date known to be progression-free for those censored. Patient demographics are summarized in **Supplementary Table 1**.

**Tissue microarray (TMA) and immunohistochemistry (IHC)**: TMAs were constructed as described previously (1). Briefly, triplicate tissue cores with a diameter of 0.6 mm were taken from the tumour and arrayed into a recipient paraffin block using a tissue puncher/arrayer (Beecher Instruments, Silver Spring, MD, USA), as previously described (Kononen *et al*, 1998). Four micron sections of the tissue array block were cut and placed on Surgipath X-tra Adhesive microscope slides (Leica Microsystems) for immunohistochemical staining. Immunohistochemical staining was performed using Novocastra Novolink polymer detection system according to manufacturer instructions (Leica Microsystems, Newcastle, UK). Pre-treatment of TMA sections was performed with citrate buffer (pH 6.0, 20 min, Microwave). For polβ IHC staining, the TMA sections were incubated for 60 min at room temperature with 1:200 dilutions of anti-pol β rabbit polyclonal antibody (clone ab26343, Abcam). Specificity of polβ antibody was confirmed using immunofluorescence staining in control and polβ knock-out A2780 and A2780cis cells (see pre-clinical study methods for more details, **supplementary Figure S3D**). For PARG IHC staining, the TMA sections were incubated for 60 min at room temperature with 1:100 dilutions of anti-PARG rabbit polyclonal antibody (catalog no: PA514158, Thermofisher). Negative controls with no primary antibody were included in each run. Cases with multiple cores were scored and the average was used as the final score.

**Evaluation of immune staining:** Whole field inspection of the core was scored and the subcellular localisation of each marker was identified (nuclear, cytoplasm, cell membrane). Intensities of subcellular compartments were each assessed and grouped as follows: 0 = no staining, 1 = weak staining, 2 = moderate staining, 3 = strong staining. The percentage of tumour cells in each category was estimated (0–100%). H-score (range 0–300) was calculated by multiplying the intensity of staining and the percentage of staining. Low/negative nuclear polβ (polβ-) expression was defined by median H-score of ≤180. Low/negative cytoplasmic PARG expression was defined by X-tile H-score of ≤105. Not all cores within the TMA were suitable for IHC analysis due to missing cores or absence of tumour cells.

**Statistical analysis:** This was performed using SPSS, version 22 (Chicago, IL, USA) for Windows. Association with clinical and pathological parameters using categorised data was examined using Chi-squared test. All tests were 2-tailed. Survival rates were determined using Kaplan–Meier method and compared by the log-rank test. All analyses were conducted using Statistical Package for the Social Sciences (SPSS, version 22; Chicago, IL, USA) software for windows. P value of - <0.05 was identified as statistically significant. This work was approved by the Nottingham Research Ethics Committee.

***Polβ* mRNA expression and human epithelial ovarian cancers:** Prognostic significance of *polβ* mRNA expression was investigated in publically available ovarian tumour gene expression data sets (<http://kmplot.com/analysis/index.php?p=service&cancer=ovar>) (2). A test cohort of 107 tumors (3), validation cohort 1 of 285 tumors (4) and validation cohort 2 (TCGA) of 465 tumors (<http://cancergenome.nih.gov/>) (5) that received platinum based chemotherapy were evaluated in this analysis.

**Compounds and antibodies:** The antibodies used in this study are as follows**:** polβ, Poly (ADP-Ribose) polymer, p-CHK1, E-cadherin, TGF-β, MMP-9 antibodies were purchased from Abcam, UK. PARG, N-cadherin antibodies were purchased from ThermoFisher, UK. ATR, PARP-1 antibodies were obtained from Cell Signalling, USA. Histone H2AX phosphorylated at Ser^139^ (γH2AX) were purchased from Millipore, UK. Calcein AM and Ethidium homodimer -1 were purchased from ThermoFisher, UK. Cisplatin solution (1 mg/ml) was obtained from the Department of Pharmacy, Nottingham University Hospitals, Nottingham,UK. Olaparib (AZD2281) was kindly provided by AstraZeneca Pharmaceuticals. PARG inhibitor, PDD00017273 was purchased from Selleckchem, UK. NSC666719 [4-chloro-5-methyl-N-[5-(naphthalen-2-ylamino)-1H1,2,4-triazol-3-yl]-2-sulfanylbenzenesulfonamide] (polβ small molecule inhibitor) was obtained from the Developmental Therapeutics Program of the National Cancer Institute of the National Institutes of Health (DTP, NCI-NIH) that has been described in our previous studies (6-8). Pamoic acid (polβ small molecule inhibitor) (9) was purchased from Selleckchem, UK. Talazoparib was purchased from Selleckchem, UK.

**Cell lines and culture:** A2780 (platinum sensitive) and A2780cis (platinum resistant), PEO1 (BRCA2-deficient), PE04 (BRCA2-proficient) were purchased from American Type Culture Collection (ATCC, Manassas, USA). Cells were cultured in RPMI medium supplemented with 10% FBS and 1% penicillin/streptomycin. To maintain cisplatin resistance in A2780cis, cells were exposed to 1 μM cisplatin every 2-3 passages. BRCA2-deficient HeLa SilenciX cells and controls BRCA2-proficient HeLa SilenciX cells were purchased from Tebu-Bio ([www.tebu-bio.com](http://www.tebu-bio.com/)). SilenciX cells were grown in Dulbecco's Modified Eagle's Medium supplemented with 10% FBS, 1% penicillin/streptomycin, and 125 μg/mL hygromycin B.

All cell lines were tested for mycoplasma contamination on a three monthly basis. Cell lines were authenticated by STR profiling.

**Targeted next generation sequencing and bioinformatics:** Genomic DNA was extracted from cell lines using the PicoPure™ DNA Extraction Kit (Thermofisher,UK). Targeted next generation sequencing was used to identify genomic variants in platinum sensitive (A2780, PE01) and platinum resistant derivatives (A2780cis, PE04). The SureSelect All Exon V5 kit (Agilent Technologies) was used to enrich for protein coding regions and sequencing performed using an Illumina NextSeq500 sequencer with paired end reads (150bp) and a minimum of 88million reads generated per sample. Raw reads were fastq formatted. Contaminating adapter sequences and low-quality sequences were processed using Skewer (10). Quality processed reads were aligned to the  HG19 reference genome using BWA (11), duplicate alignments identified and processed using PicardTools, and realignment completed using the Abra assembly based realigner (12) to enhance detection of insertion/deletion variants. Variant calling and filtering was completed using Samtools/Bcftools (v1.3.1) (13). Variants, in variant call format (VCF), associated with Platinum resistance were identified using vcf tools (14). Variants were annotated and functional significance assessed using the Ensembl Variant Effect Predictor tool (15). Library preparation and sequencing was conducted by Source Biosciences (Nottingham, UK). Genes affecting the Polβ mammalian interactome were identified using the BioGrid database (16). The gene list from the polβ interactome (n=46) was interrogated in the A2780cis and PEO4 exome sequencing dataset to identify gene variants. The biological function of each gene variant was identified using Webgestalt gene set analysis tool, (17)and ORA (overrepresentation enrichment analysis based on gene ontology (GO)-annotated biological processes) (18). The ratio of observed versus expected number of genes in the category was recorded for each significant category using the enrichment ratio (R) scores.

**Polβ knockdown using siRNAs:** Polβ siRNA constructs as well as negative scrambled control were purchased from Invitrogen, UK. Cells were seeded at 60-70% confluency in T25 flasks overnight. Lipofectamine 3000 reagent (Invitrogen, UK) was used to transfect the cells as per the manufacturer’s protocol. Polβ knockdown was evaluated by western blotting at various time points after transfection (days 3, 5 and 7).

**Generation of polβ knockouts using CRISPR/Cas-9 system:** A2780 and A2780cis were transfected with oligonucleotides carrying gRNA silencing polβ cloned in Plv-U6g-EPCG plasmid (Sigma, UK). Briefly, cells were seeded at 50-60% confluency in 6-well plates overnight. Cells were transfected with 2-3 µg of DNA using Lipofectamine 3000 (Invitrogen, UK) in Opti-MEM medium. Puromycin (10 µg/ml) was used for the selection of desired clones for 14 days.

**Clonogenic assays:** For A2780 and A2780cis ovarian cell lines200 cells/ per well were seeded overnight in 6-well plates. For HeLa control and HeLa_BRCA2_KD cells 250 cells/ per well were seeded in 6-well plates. For PE01/ PE04 ovarian cell lines, 1,000 cells of PE01 and 300 cells of PE04 were seeded overnight Platting efficiency for each cell line were determined before hands by plating different cell densities for each line and incubating them at 37^0^c for 14 days. Platting efficiency were calculated using the formula (number of colonies formed/ number of cells incubated) x100. For testing drugs’ sensitivity, cells were plated overnight, then compounds were added at the indicated concentrations. The plates were left in the incubator for 14 days, after incubation colonies were washed with PBS, fixed and stained with crystal violet, acetic acid and methanol mixture and counted. Survival fraction (SF) were calculated using the formula SF = no. of colonies formed after treatment/no. of cells seeded x platting efficiency. Number of colonies counted were normalised relative to the count of untreated wells which were considered as 100% survival.

**Cell proliferation assays:** A2780 and A2780cis control and polβ KO cells (100 cells/well)

were seeded in 96-well plates and left to adhere overnight. The following day cells were treated with 30 μM curcumin for 24 hours or left untreated. Then cells were treated with Cisplatin at the indicated concentration for five days. Cell viability was measured by CellTiter cell proliferation assay (MTS) (Promega, UK). For validation of Mitomycin C sensitivity, A2780 and A2780cis control and polβ KO cells were plated overnight. Cells were treated with the indicated concentrations of Mitomycin C. Cell viability was measured by CellTiter cell proliferation assa (MTS) (Promega, UK).

**Cell cycle and Apoptosis analysis by flow cytometry:** Cells were plated overnight then treated with the tested inhibitors at the specified concentrations or left untreated. After 24 h Cells were trypsinized and washed with ice cold PBS, and fixed in 70% ethanol for at least 30 min. After removal of the fixative ethanol by centrifugation cells were stained with phosphor-Histone (γH2AX) Ser139 for double-strand breaks detection. For cell cycle progression, cells were treated with RNase and stained with 10 µg/ml propidium iodide (Sigma Aldrich) in PBS. For Apoptosis detection, cells were collected and analysed using annexinV detection kit (BD biosciences). Samples were analysed on FC500 flow-cytometer (Beckman Coulter) and data were analysed using Weasel software.

**Invasion and migration assays:** Cells were seeded in the upper chamber of polycarbonate membrane inserts (8 µm pore size), (Cell Biolabs, UK) in serum-free medium and left to invade toward 10% serum containing medium for 24 h. Then, the medium containing non-invasive cells were aspirated from the inserts and the inner side was washed with distilled water, then stained with crystal violet for 10 min. Cells were extracted, and 100 µL from each sample were transferred to 96-well microtiter plate for measuring OD at 560 nm. For migration assays, cells were seeded in 96-well plate containing hydrogel spot non-migratory area, left to adhere overnight and then hydrogel area was digested and cells were left to migrate for 20 h. Then the wells were washed three-times, fixed and stained as per manufacturer’s protocol. Cell migration images were analysed by ImageJ software.

**qRT-PCR analysis of epithelial-mesenchymal transition (EMT) gene expression:** Real time PCR was performed using RT^2^ Profiler EMT PCR Array for 86 EMT genes. The data was analysed as per manufacturer’s recommendations (<https://www.qiagen.com/us/shop/genes-and-pathways/data-analysis-center-overview-page/>). HPRT1 was used for normalization of the data. All experiments were performed in duplicate.

**Western Blotting:** Protein samples were prepared by lysing cells in RIPA buffer (Sigma-Aldrich) containing protease inhibitor (Sigma-Aldrich) and phosphatase inhibitor cocktail 2 and 3 (Sigma-Aldrich). Protein quantification was performed using BCA colorimetric kit (ThermoFisher, UK). Membranes were incubated with primary antibodies (4°C, overnight), washed and later incubated with infrared dye-labeled secondary antibodies (Li-cor) [IRDye 800CW Donkey Anti-Rabbit IgG (H+L) and IRDye 680CW Donkey Anti-Mouse IgG (H+L)] in the dilution of 1:10,000 for 60 min. Protein detection and quantification was determined by scanning the membranes on Licor-Odyssey's Scanner (Licor, Biosciences) at the predefined intensity fluorescence.

**Confocal microscopy:** Cells were seeded on the cover slips overnight, then treated with PDP00017273 for the indicated time-points. The cells were fixed with 4% (w/v) paraformaldehyde for 30 min, permeabilized with 0.1% (w/v) Triton X100 (ThermoFisher) for 30 min and blocked with 3% (w/v) BSA for 1 h. Cells were incubated with anti-Poly(ADP-Ribose) polymer antibody (Abcam, ab14459) or with anti- 53BP1 (Cell Signalling, catalogue no. 4937S) and anti γH2AX (Merck millipore clone JBW301) overnight at 4°C. Slides were prepared in duplicates. Imaging was carried out using Leica SP2 confocal laser scanning microscope. For analysis a minimum of 100 cells per slide were counted. Nuclear fluorescence was quantified for γH2AX using ImageJ software as well as for 53BP1 and Poly (ADP-Ribose) polymer nuclear fluorescence. Values were plotted in GraphPad Prism 7.

**PAR quantification by ELISA:** PAR levels were also evaluated using an ELISA kit (Cell Biolabs, catalog number XDN-5114) as per manufacturer’s protocol. The ELISA kit is a sandwich ELISA for the quantification of PAR.

**PARG ELISA assay:** To measure endogenous PARG enzymatic activity A2780 control, A2780 _polβ_KO cells, A2780cis control A2780 _polβ_KO cells were lysed in RIPA buffer. Protein extracted were loaded into PARG antibody pre-coated 96-well ELISA plate (Abbexa, abx385260) according to the manufacturer’s protocol.

**NAD+ level quantification assay:** NAD+ quantification in polβ_KO and control cells following PARGi (25 µM) treatment for 16 h using the NAD+/NADH colorimetric assay kit (catalog ID: ab221821, Abcam, UK) as per manufacturer's protocol. Briefly, following PARGi treatment cells were collected by trypsinization and washed with ice-cold PBS, then NAD+ was extracted in acidic condition using 0.5 M percloric acid. Protein content in the samples was quantified via BCA protein quantification kit (ThermoFisher, UK). Extracts were diluted in NAD+ dilution buffer and OD were measured at 450 nm as per the supplier protocol in TECAN microplate reader.

**Generation of 3D spheroids:** Cells **(**4x10^4^) were seeded in ultra-low attachment 6-well plates using promo cell tumour spheres medium. After that cells were left to form spheres for 3-weeks. Spheroids were treated with the indicated inhibitors for 48 h. Then, spheres were fixed with formaldehyde (3.7%, w/v) and stained with 2 µM calcein AM and 1.5 µM ethidiumhomodimer-1. Imaging was carried out using Leica SP2 confocal laser scanning microscope. Images were analyzed by ImageJ software.

**References**

1. Kononen J, Bubendorf L, Kallioniemi A, Barlund M, Schraml P, Leighton S, et al. Tissue microarrays for high-throughput molecular profiling of tumor specimens. Nat Med **1998**;4:844-7

2. Gyorffy B, Lanczky A,Szallasi Z. Implementing an online tool for genome-wide validation of survival-associated biomarkers in ovarian-cancer using microarray data from 1287 patients. Endocr Relat Cancer **2012**;19:197-208

3. Mateescu B, Batista L, Cardon M, Gruosso T, de Feraudy Y, Mariani O, et al. miR-141 and miR-200a act on ovarian tumorigenesis by controlling oxidative stress response. Nat Med **2011**;17:1627-35

4. Tothill RW, Tinker AV, George J, Brown R, Fox SB, Lade S, et al. Novel molecular subtypes of serous and endometrioid ovarian cancer linked to clinical outcome. Clin Cancer Res **2008**;14:5198-208

5. Denkert C, Budczies J, Darb-Esfahani S, Gyorffy B, Sehouli J, Konsgen D, et al. A prognostic gene expression index in ovarian cancer - validation across different independent data sets. J Pathol **2009**;218:273-80

6. Jaiswal AS, Banerjee S, Aneja R, Sarkar FH, Ostrov DA,Narayan S. DNA polymerase beta as a novel target for chemotherapeutic intervention of colorectal cancer. PLoS One **2011**;6:e16691

7. Jaiswal AS, Banerjee S, Panda H, Bulkin CD, Izumi T, Sarkar FH, et al. A novel inhibitor of DNA polymerase beta enhances the ability of temozolomide to impair the growth of colon cancer cells. Mol Cancer Res **2009**;7:1973-83

8. Jaiswal AS, Panda H, Law BK, Sharma J, Jani J, Hromas R, et al. NSC666715 and Its Analogs Inhibit Strand-Displacement Activity of DNA Polymerase beta and Potentiate Temozolomide-Induced DNA Damage, Senescence and Apoptosis in Colorectal Cancer Cells. PLoS One **2015**;10:e0123808

9. Hazan C, Boudsocq F, Gervais V, Saurel O, Ciais M, Cazaux C, et al. Structural insights on the pamoic acid and the 8 kDa domain of DNA polymerase beta complex: towards the design of higher-affinity inhibitors. BMC Struct Biol **2008**;8:22

10. Jiang H, Lei R, Ding SW,Zhu S. Skewer: a fast and accurate adapter trimmer for next-generation sequencing paired-end reads. BMC Bioinformatics **2014**;15:182

11. Li H,Durbin R. Fast and accurate long-read alignment with Burrows-Wheeler transform. Bioinformatics **2010**;26:589-95

12. Mose LE, Wilkerson MD, Hayes DN, Perou CM,Parker JS. ABRA: improved coding indel detection via assembly-based realignment. Bioinformatics **2014**;30:2813-5

13. Li H, Handsaker B, Wysoker A, Fennell T, Ruan J, Homer N, et al. The Sequence Alignment/Map format and SAMtools. Bioinformatics **2009**;25:2078-9

14. Danecek P, Auton A, Abecasis G, Albers CA, Banks E, DePristo MA, et al. The variant call format and VCFtools. Bioinformatics **2011**;27:2156-8

15. McLaren W, Gil L, Hunt SE, Riat HS, Ritchie GR, Thormann A, et al. The Ensembl Variant Effect Predictor. Genome Biol **2016**;17:122

16. Oughtred R, Stark C, Breitkreutz BJ, Rust J, Boucher L, Chang C, et al. The BioGRID interaction database: 2019 update. Nucleic Acids Res **2019**;47:D529-D41

17. Wang J, Vasaikar S, Shi Z, Greer M,Zhang B. WebGestalt 2017: a more comprehensive, powerful, flexible and interactive gene set enrichment analysis toolkit. Nucleic Acids Res **2017**;45:W130-W7

18. The Gene Ontology C. Expansion of the Gene Ontology knowledgebase and resources. Nucleic Acids Res **2017**;45:D331-D8
