# Supplementary material for: Molecular disruption of DNA polymerase β for platinum sensitisation and synthetic lethality in epithelial ovarian cancers
Source: Oncogene. 2021 Mar 5;40(14):2496–508. doi: 10.1038/s41388-021-01710-y (PMC8032555; doi:10.1038/s41388-021-01710-y)
Supplement: Supplementary file 2 — Supplementary Tables [file 41388_2021_1710_MOESM2_ESM.docx]

**Supplementary Table S1:** Polβ expression and clinico-pathological associations.

|  | **Polβ (Low) N (%)** | **Polβ (High) N (%)** | **P-value** |
| --- | --- | --- | --- |
| ***Pathological Type*** |  |  | ***0.001*** |
| Serous cystadenocarcinoma | 113 (48.5) | 120 (51.5) |  |
| Mucinous cystadenocarcinoma | 32 (74.4) | 11 (25.6) |  |
| Endometrioid | 49 (69.0) | 22 (31.0) |  |
| Clear cell carcinoma | 26 (70.3) | 11 (29.7) |  |
| Mixed | 6 (50.0) | 6 (50.0) |  |
| Other | 4 (33.3) | 8 (66.7) |  |
| ***FIGO Stage*** |  |  | ***0.003*** |
| I | 101 (67.3) | 49 (32.7) |  |
| II | 25 (41.0) | 36 (59.0) |  |
| III | 87 (52.4) | 79 (47.6) |  |
| IV | 15 (55.6) | 12 (44.4) |  |
| ***Tumour Grade*** |  |  | 0.072 |
| G1 | 30 (61.2) | 19 (38.8) |  |
| G2 | 45 (64.3) | 25 (35.7) |  |
| G3 | 124 (50.4) | 122 (49.6) |  |
| ***Surgical Optimal Debulking*** |  |  | ***0.023*** |
| Optimally Debulked | 180 (60.2) | 119 (39.8) |  |
| Not Optimally Debulked | 43 (46.7) | 49 (53.3) |  |
| ***Platinum sensitivity*** |  |  | *0.076* |
| Sensitive | 157 (52.0) | 145 (48.0) |  |
| Resistant | 25 (39.7) | 38 (60.3) |  |

**Supplementary Table S2**: Polβ interactome genes harbouring variants in platinum resistant A2780cis and PEO4 cell lines compared to platinum sensitive A2780 and PEO1 cell lines.

| **Ensembl** | **Location** | **Allele** | **Consequence** | **Codons** | **Codons** | **Existing_variation** | **Impact** | **Gene** |
| --- | --- | --- | --- | --- | --- | --- | --- | --- |
| ENSG00000100823 | 14:20928789-20928789 | T | downstream_gene_variant | - | - | rs1208225210 | MODIFIER | *APEX1* |
| ENSG00000137074 | 9:32974372-32974372 | T | intron_variant | - | - | rs4878522 | MODIFIER | *APTX* |
| ENSG00000137074 | 9:33026572-33026572 | C | upstream_gene_variant | - | - | rs20583 | MODIFIER | *APTX* |
| ENSG00000137074 | 9:33026638-33026638 | A | upstream_gene_variant | - | - | rs2274769 | MODIFIER | *APTX* |
| ENSG00000248098 | 19:41928867-41928870 | CCCC | intron_variant | - | - | rs3217385 | MODIFIER | *BCKDHA* |
| ENSG00000083123 | 6:80878590-80878590 | G | splice_acceptor_variant | - | - | CS1512877 | HIGH | *BCKDHB* |
| ENSG00000083123 | 6:80910622-80910634 | TTTTTTTTTT | intron_variant | - | - | rs374325532 | MODIFIER | *BCKDHB* |
| ENSG00000100393 | 22:41545024-41545038 | TTTTTTTTTTT | splice_region_variant,intron_variant | - | - | rs747710183 | LOW | *EP300* |
| ENSG00000100393 | 22:41546004-41546004 | G | synonymous_variant | P | ccA/ccG | - | LOW | *EP300* |
| ENSG00000136273 | 7:47898372-47898372 | A | intron_variant,NMD_transcript_variant | - | - | rs146609164 | MODIFIER | *HUS1* |
| ENSG00000136273 | 7:47944799-47944799 | T | intron_variant,NMD_transcript_variant | - | - | - | MODIFIER | *HUS1* |
| ENSG00000126777 | 14:56117914-56117914 | C | intron_variant | - | - | - | MODIFIER | *KTN1* |
| ENSG00000005156 | 17:33310339-33310345 | AAAAAAA | frameshift_variant | KK/KKX | AAAAAA/AAAAAAA | rs779414317 | HIGH | ***LIG3*** |
| ENSG00000140398 | 15:75651022-75651039 | CCACATGCTGCCCAGC  CCATACCACATGCTGCCCAGCC | downstream_gene_variant | - | - | rs149997575 | MODIFIER | *NEIL1* |
| ENSG00000172613 | 11:67161107-67161107 | A | intron_variant | - | - | rs1331639365 | MODIFIER | *RAD9A* |
| ENSG00000135250 | 7:104748407-104748422 | TTTTTTTTTTTTT | downstream_gene_variant | - | - | rs34102092 | MODIFIER | *SRPK2* |
| ENSG00000135250 | 7:104773568-104773568 | C | intron_variant | - | - | - | MODIFIER | *SRPK2* |
| ENSG00000103266 | 16:732287-732289 | C | splice_region_variant,intron_variant | - | - | rs3216838 | LOW | *STUB1* |
| ENSG00000042088 | 14:90447060-90447070 | TTTTTTTTTTT | intron_variant | - | - | rs35916493 | MODIFIER | *TDP1* |
| ENSG00000196781 | 9:84275256-84275269 | AAAAAAAAAAAA | intron_variant | - | - | rs36108203 | MODIFIER | *TLE1* |
| ENSG00000134900 | 13:103295551-103295564 | TTCTTTCTT | splice_region_variant,intron_variant | - | - | rs3831119 | LOW | *TPP2* |
| ENSG00000076248 | 12:109535511-109535511 | T | synonymous_variant | S | tcC/tcT | - | LOW | *UNG* |
| ENSG00000165392 | 8:31014936-31014936 | C | missense_variant | L/S | tTa/tCa | - | MODERATE | ***WRN*** |
| ENSG00000126215 | 14:104169572-104169572 | T | missense_variant | G/R | Gga/Aga | - | MODERATE | ***XRCC3*** |
| ENSG00000154767 | 3:14189477-14189477 | T | synonymous_variant | E | gaG/gaA | COSV53205101 | LOW | *XPC* |
| ENSG00000154767 | 3:14201182-14201182 | C | intron_variant | - | - | - | MODIFIER | *XPC* |
| ENSG00000113851 | 3:3194107-3194117 | AAAAAAAAAAA | intron_variant | - | - | rs11414437 | MODIFIER | *CRBN* |
| ENSG00000166012 | 11:93474570-93474571 | GCGTGAG | inframe_insertion | T/THA | aCa/aCTCACGCa | rs5793653 | MODERATE | ***TAF1D*** |

**Supplementary Table S3: List of EMT PCR array genes.**

| **Position** | **Unigene** | **Refseq** | **Symbol** | **Description** | **Name** | **RT2 Catalog** |
| --- | --- | --- | --- | --- | --- | --- |
| A01 | Hs.502756 | NM_024060 | AHNAK | AHNAK nucleoprotein | AHNAKRS | PPH18485A |
| A02 | Hs.525622 | NM_005163 | AKT1 | V-akt murine thymoma viral oncogene homolog 1 | AKT/CWS6/PKB/PKB-ALPHA/PRKBA/RAC/RAC-ALPHA | PPH00088B |
| A03 | Hs.1274 | NM_006129 | BMP1 | Bone morphogenetic protein 1 | OI13/PCOLC/PCP/PCP2/TLD | PPH00515B |
| A04 | Hs.73853 | NM_001200 | BMP2 | Bone morphogenetic protein 2 | BDA2/BMP2A | PPH00549C |
| A05 | Hs.473163 | NM_001719 | BMP7 | Bone morphogenetic protein 7 | OP-1 | PPH00527A |
| A06 | Hs.490203 | NM_004342 | CALD1 | Caldesmon 1 | CDM/H-CAD/HCAD/L-CAD/LCAD/NAG22 | PPH21139A |
| A07 | Hs.731383 | NM_018584 | CAMK2N1 | Calcium/calmodulin-dependent protein kinase II inhibitor 1 | PRO1489 | PPH14215B |
| A08 | Hs.212332 | NM_001233 | CAV2 | Caveolin 2 | CAV | PPH12406B |
| A09 | Hs.461086 | NM_004360 | CDH1 | Cadherin 1, type 1, E-cadherin (epithelial) | Arc-1/CD324/CDHE/ECAD/LCAM/UVO | PPH00135F |
| A10 | Hs.464829 | NM_001792 | CDH2 | Cadherin 2, type 1, N-cadherin (neuronal) | CD325/CDHN/CDw325/NCAD | PPH00636F |
| A11 | Hs.489142 | NM_000089 | COL1A2 | Collagen, type I, alpha 2 | OI4 | PPH01918B |
| A12 | Hs.443625 | NM_000090 | COL3A1 | Collagen, type III, alpha 1 | EDS4A | PPH00439F |
| B01 | Hs.445827 | NM_000393 | COL5A2 | Collagen, type V, alpha 2 | EDSC | PPH00781A |
| B02 | Hs.712929 | NM_001904 | CTNNB1 | Catenin (cadherin-associated protein), beta 1, 88kDa | CTNNB/MRD19/armadillo | PPH00643F |
| B03 | Hs.95612 | NM_004949 | DSC2 | Desmocollin 2 | ARVD11/CDHF2/DG2/DGII/III/DSC3 | PPH10581B |
| B04 | Hs.519873 | NM_004415 | DSP | Desmoplakin | DCWHKTA/DP/DPI/DPII | PPH17443A |
| B05 | Hs.488293 | NM_005228 | EGFR | Epidermal growth factor receptor | ERBB/ERBB1/HER1/NISBD2/PIG61/mENA | PPH00138B |
| B06 | Hs.118681 | NM_001982 | ERBB3 | V-erb-b2 erythroblastic leukemia viral oncogene homolog 3 (avian) | ErbB-3/HER3/ B3 | PPH00463B |
| B07 | Hs.744830 | NM_000125 | ESR1 | Estrogen receptor 1 | ER/ESR/ESRA/ESTRR/Era/NR3A1 | PPH01001A |
| B08 | Hs.517293 | NM_016946 | F11R | F11 receptor | CD321/JAM/JAM1/JAMA/JCAM/KAT/PAM-1 | PPH02605A |
| B09 | Hs.1690 | NM_005130 | FGFBP1 | Fibroblast growth factor binding protein 1 | FGF-BP/FGF-BP1/FGFBP/FGFBP-1/HBP17 | PPH07093A |
| B10 | Hs.203717 | NM_002026 | FN1 | Fibronectin 1 | CIG/ED-B/FINC/FN/FNZ/GFND/GFND2/LETS/MSF | PPH00143B |
| B11 | Hs.436448 | NM_005251 | FOXC2 | Forkhead box C2 (MFH-1, mesenchyme forkhead 1) | FKHL14/LD/MFH-1/MFH1 | PPH01971A |
| B12 | Hs.173859 | NM_003507 | FZD7 | Frizzled family receptor 7 | FzE3 | PPH02420B |
| C01 | Hs.83381 | NM_004126 | GNG11 | Guanine nucleotide binding protein (G protein), gamma 11 | GNGT11 | PPH02836C |
| C02 | Hs.440438 | NM_173849 | GSC | Goosecoid homeobox | SAMS | PPH01920A |
| C03 | Hs.445733 | NM_002093 | GSK3B | Glycogen synthase kinase 3 beta | - | PPH00787C |
| C04 | Hs.462998 | NM_001552 | IGFBP4 | Insulin-like growth factor binding protein 4 | BP-4/HT29-IGFBP/IBP4/IGFBP-4 | PPH00286B |
| C05 | Hs.81134 | NM_000577 | IL1RN | Interleukin 1 receptor antagonist | DIRA/ICIL-1RA/IL-1RN/IL-1ra/IL-1ra3/IL1F3/IL1RA/IRAP/MVCD4 | PPH00555G |
| C06 | Hs.706355 | NM_004517 | ILK | Integrin-linked kinase | HEL-S-28/ILK-1/ILK-2/P59/p59ILK | PPH00737F |
| C07 | Hs.505654 | NM_002205 | ITGA5 | Integrin, alpha 5 (fibronectin receptor, alpha polypeptide) | CD49e/FNRA/VLA-5/VLA5A | PPH00176C |
| C08 | Hs.436873 | NM_002210 | ITGAV | Integrin, alpha V (vitronectin receptor, alpha polypeptide, antigen CD51) | CD51/MSK8/VNRA/VTNR | PPH00628C |
| C09 | Hs.643813 | NM_002211 | ITGB1 | Integrin, beta 1 (fibronectin receptor, beta polypeptide, antigen CD29 includes MDF2, MSK12) | CD29/FNRB/GPIIA/MDF2/MSK12/VLA-BETA/VLAB | PPH00650B |
| C10 | Hs.224012 | NM_000214 | JAG1 | Jagged 1 | AGS/AHD/AWS/CD339/HJ1/JAGL1 | PPH06022B |
| C11 | Hs.654380 | NM_000526 | KRT14 | Keratin 14 | CK14/EBS3/EBS4/K14/NFJ | PPH02389A |
| C12 | Hs.654568 | NM_002276 | KRT19 | Keratin 19 | CK19/K19/K1CS | PPH01004E |
| D01 | Hs.411501 | NM_005556 | KRT7 | Keratin 7 | CK7/K2C7/K7/SCL | PPH08502E |
| D02 | Hs.335079 | NM_005909 | MAP1B | Microtubule-associated protein 1B | FUTSCH/MAP5/PPP1R102 | PPH02398A |
| D03 | Hs.513617 | NM_004530 | MMP2 | Matrix metallopeptidase 2 (gelatinase A, 72kDa gelatinase, 72kDa type IV collagenase) | CLG4/CLG4A/MMP-2/MMP-II/MONA/TBE-1 | PPH00151B |
| D04 | Hs.375129 | NM_002422 | MMP3 | Matrix metallopeptidase 3 (stromelysin 1, progelatinase) | CHDS6/MMP-3/SL-1/STMY/STMY1/STR1 | PPH00235F |
| D05 | Hs.297413 | NM_004994 | MMP9 | Matrix metallopeptidase 9 (gelatinase B, 92kDa gelatinase, 92kDa type IV collagenase) | CLG4B/GELB/MANDP2/MMP-9 | PPH00152E |
| D06 | Hs.713679 | NM_002444 | MSN | Moesin | HEL70 | PPH13452B |
| D07 | Hs.517973 | NM_002447 | MST1R | Macrophage stimulating 1 receptor (c-met-related tyrosine kinase) | CD136/CDw136/PTK8/RON | PPH07170B |
| D08 | Hs.370414 | NM_018055 | NODAL | Nodal homolog (mouse) | HTX5 | PPH01944B |
| D09 | Hs.495473 | NM_017617 | NOTCH1 | Notch 1 | AOS5/AOVD1/TAN1/hN1 | PPH00526C |
| D10 | Hs.533657 | NM_015901 | NUDT13 | Nudix (nucleoside diphosphate linked moiety X)-type motif 13 | - | PPH20529A |
| D11 | Hs.592605 | NM_002538 | OCLN | Occludin | BLCPMG/PPP1R115 | PPH02571B |
| D12 | Hs.509067 | NM_002609 | PDGFRB | Platelet-derived growth factor receptor, beta polypeptide | CD140B/IBGC4/IMF1/JTK12/PDGFR/PDGFR-1/PDGFR1 | PPH00477C |
| E01 | Hs.170473 | NM_016445 | PLEK2 | Pleckstrin 2 | - | PPH13458F |
| E02 | Hs.570455 | NM_015704 | DESI1 | PPPDE peptidase domain containing 2 | D15Wsu75e/DESI2/DJ347H13.4/DeSI-1/FAM152B/PPPDE2 | PPH23010A |
| E03 | Hs.395482 | NM_005607 | PTK2 | PTK2 protein tyrosine kinase 2 | FADK/FAK/FAK1/FRNK/PPP1R71/p125FAK/pp125FAK | PPH02827A |
| E04 | Hs.227777 | NM_003463 | PTP4A1 | Protein tyrosine phosphatase type IVA, member 1 | HH72/PRL-1/PRL1/PTP(CAAX1)/PTPCAAX1 | PPH14671C |
| E05 | Hs.413812 | NM_006908 | RAC1 | Ras-related C3 botulinum toxin substrate 1 (rho family, small GTP binding protein Rac1) | MIG5/Rac-1/TC-25/p21-Rac1 | PPH00733F |
| E06 | Hs.78944 | NM_002923 | RGS2 | Regulator of G-protein signaling 2, 24kDa | G0S8 | PPH02231A |
| E07 | Hs.414795 | NM_000602 | SERPINE1 | Serpin peptidase inhibitor, clade E (nexin, plasminogen activator inhibitor type 1), member 1 | PAI/PAI-1/PAI1/PLANH1 | PPH00215F |
| E08 | Hs.652307 | NM_003616 | GEMIN2 | Survival of motor neuron protein interacting protein 1 | SIP1/SIP1-delta | PPH10871A |
| E09 | Hs.12253 | NM_005901 | SMAD2 | SMAD family member 2 | JV18/JV18-1/MADH2/MADR2/hMAD-2/hSMAD2 | PPH01949F |
| E10 | Hs.48029 | NM_005985 | SNAI1 | Snail homolog 1 (Drosophila) | SLUGH2/SNA/SNAH/SNAIL/SNAIL1/dJ710H13.1 | PPH02459B |
| E11 | Hs.360174 | NM_003068 | SNAI2 | Snail homolog 2 (Drosophila) | SLUG/SLUGH1/SNAIL2/WS2D | PPH02475A |
| E12 | Hs.673548 | NM_178310 | SNAI3 | Snail homolog 3 (Drosophila) | SMUC/SNAIL3/ZNF293/Zfp293 | PPH15155C |
| F01 | Hs.376984 | NM_006941 | SOX10 | SRY (sex determining region Y)-box 10 | DOM/PCWH/WS2E/WS4/WS4C | PPH02458C |
| F02 | Hs.111779 | NM_003118 | SPARC | Secreted protein, acidic, cysteine-rich (osteonectin) | BM-40/ON | PPH01175A |
| F03 | Hs.313 | NM_000582 | SPP1 | Secreted phosphoprotein 1 | BNSP/BSPI/ETA-1/OPN | PPH00582E |
| F04 | Hs.463059 | NM_003150 | STAT3 | Signal transducer and activator of transcription 3 (acute-phase response factor) | ADMIO/APRF/HIES | PPH00708F |
| F05 | Hs.61635 | NM_012449 | STEAP1 | Six transmembrane epithelial antigen of the prostate 1 | PRSS24/STEAP | PPH02268C |
| F06 | Hs.371282 | NM_003200 | TCF3 | Transcription factor 3 (E2A immunoglobulin enhancer binding factors E12/E47) | E2A/E47/ITF1/TCF-3/VDIR/bHLHb21 | PPH06916G |
| F07 | Hs.742885 | NM_003199 | TCF4 | Transcription factor 4 | E2-2/ITF-2/ | PPH02770A |
| F08 | Hs.438231 | NM_006528 | TFPI2 | Tissue factor pathway inhibitor 2 | PP5/REF1/TFPI-2 | PPH02580A |
| F09 | Hs.645227 | NM_000660 | TGFB1 | Transforming growth factor, beta 1 | CED/DPD1/LAP/TGFB/TGFbeta | PPH00508A |
| F10 | Hs.133379 | NM_003238 | TGFB2 | Transforming growth factor, beta 2 | LDS4/TGF-beta2 | PPH00524B |
| F11 | Hs.713281 | NM_003239 | TGFB3 | Transforming growth factor, beta 3 | ARVD/ARVD1/RNHF/TGF-beta3 | PPH00531F |
| F12 | Hs.522632 | NM_003254 | TIMP1 | TIMP metallopeptidase inhibitor 1 | CLGI/EPA/EPO/HCI/TIMP | PPH00771C |
| G01 | Hs.598100 | NM_003692 | TMEFF1 | Transmembrane protein with EGF-like and two follistatin-like domains 1 | C9orf2/CT120.1/H7365/TR-1 | PPH17229A |
| G02 | Hs.118552 | NM_178031 | TMEM132A | Transmembrane protein 132A | GBP/HSPA5BP1 | PPH11409A |
| G03 | Hs.364544 | NM_014399 | TSPAN13 | Tetraspanin 13 | NET-6/NET6/TM4SF13 | PPH17916A |
| G04 | Hs.66744 | NM_000474 | TWIST1 | Twist homolog 1 (Drosophila) | ACS3/BPES2/BPES3/CRS/CRS1/CSO/SCS/TWIST/bHLHa38 | PPH02132A |
| G05 | Hs.643801 | NM_004385 | VCAN | Versican | CSPG2/ERVR/GHAP/PG-M/WGN/WGN1 | PPH06098D |
| G06 | Hs.455493 | NM_003380 | VIM | Vimentin | CTRCT30/HEL113 | PPH00417F |
| G07 | Hs.459790 | NM_033305 | VPS13A | Vacuolar protein sorting 13 homolog A (S. cerevisiae) | CHAC/CHOREIN | PPH09443A |
| G08 | Hs.108219 | NM_004626 | WNT11 | Wingless-type MMTV integration site family, member 11 | HWNT11 | PPH02399C |
| G09 | Hs.643085 | NM_003392 | WNT5A | Wingless-type MMTV integration site family, member 5A | hWNT5A | PPH02410A |
| G10 | Hs.306051 | NM_032642 | WNT5B | Wingless-type MMTV integration site family, member 5B | - | PPH02447C |
| G11 | Hs.124503 | NM_030751 | ZEB1 | Zinc finger E-box binding homeobox 1 | AREB6/ | PPH01922A |
| G12 | Hs.34871 | NM_014795 | ZEB2 | Zinc finger E-box binding homeobox 2 | HSPC082/SIP-1/SIP1/SMADIP1/ZFHX1B | PPH09021B |
| H01 | Hs.520640 | NM_001101 | ACTB | Actin, beta | BRWS1/PS1TP5BP1 | PPH00073G |
| H02 | Hs.534255 | NM_004048 | B2M | Beta-2-microglobulin | - | PPH01094E |
| H03 | Hs.592355 | NM_002046 | GAPDH | Glyceraldehyde-3-phosphate dehydrogenase | G3PD/GAPD/HEL-S-162eP | PPH00150F |
| H04 | Hs.412707 | NM_000194 | HPRT1 | Hypoxanthine phosphoribosyltransferase 1 | HGPRT/HPRT | PPH01018C |
| H05 | Hs.546285 | NM_001002 | RPLP0 | Ribosomal protein, large, P0 | L10E/LP0/P0/PRLP0/RPP0 | PPH21138F |
| H06 | N/A | SA_00105 | HGDC | Human Genomic DNA Contamination | HIGX1A |  |
| H07 | N/A | SA_00104 | RTC | Reverse Transcription Control | RTC | PPX63340A |
| H08 | N/A | SA_00104 | RTC | Reverse Transcription Control | RTC | PPX63340A |
| H09 | N/A | SA_00104 | RTC | Reverse Transcription Control | RTC | PPX63340A |
| H10 | N/A | SA_00103 | PPC | Positive PCR Control | PPC |  |
| H11 | N/A | SA_00103 | PPC | Positive PCR Control | PPC |  |
| H12 | N/A | SA_00103 | PPC | Positive PCR Control | PPC |  |

**Supplementary Table S4:** PARG expression and clinico-pathological associations.

|  | **PARG (Low) (%)** | **PARG (High) (%)** | **P-value** |
| --- | --- | --- | --- |
| ***Pathological Type*** |  |  | ***0.011*** |
| Serous cystadenocarcinoma | 25 (41.7%) | 129(60.3%) |  |
| Mucinous cystadenocarcinoma | 11(18.3%) | 26(12.1%) |  |
| Endometrioid | 9(15.0%) | 30(14.0%) |  |
| Clear cell carcinoma | 10(16.7%) | 9(4.2%) |  |
| Mixed | 2(3.3%) | 10(4.7%) |  |
| Other | 3(5.0%) | 10(4.7%) |  |
| ***Surgical pathology Stage*** |  |  | *0.149* |
| I | 26(45.6%) | 79(38.5%) |  |
| II | 4(7.0%) | 38(18.5%) |  |
| III | 26(45.6%) | 80(3.9%) |  |
| IV | 1(1.8%) | 8(3.9%) |  |
| ***Tumour Grade*** |  |  | 0.559 |
| Low | 8(16.7%) | 29(15.4%) |  |
| Intermediate | 8(16.7%) | 45(23.9%) |  |
| High | 32(66.7%) | 114(60.6%) |  |
| ***Surgical Optimal Debulking*** |  |  | *0.134* |
| Optimally Debulked | 51(87.9%) | 152(79.2%) |  |
| Not Optimally Debulked | 7(12.1%) | 40(20.8%) |  |
| ***Platinum sensitivity*** |  |  | *0.646* |
| Sensitive | 51(89.5%) | 161(91.5%) |  |
| Resistant | 6(10.5%) | 15(8.5%) |  |

**Supplementary Table S5:** Polβ/PARG co-expression and clinico-pathological associations.

|  | **Polβ-/PARG-** | **Polβ+**  **/PARG-** | **Polβ-/PARG+** | **Polβ+**  **/PARG+** | **P-value** |
| --- | --- | --- | --- | --- | --- |
| ***Pathological Type*** |  |  |  |  | **0.017** |
| Serous cystadenocarcinoma | 17(42.5%) | 4(50.0%) | 43(54.4%) | 65(68.4%) |  |
| Mucinous cystadenocarcinoma | 6(15.0%) | 2(25.0%) | 16(20.3%) | 4(14.3%) |  |
| Endometrioid | 7(17.5%) | 0(0%) | 12(15.2%) | 11(11.6%) |  |
| Clear cell carcinoma | 7(17.5%) | 2(25.0%) | 2(2.5%) | 6(6.3%) |  |
| Mixed | 2(5%) | 0(0%) | 4(5.1%) | 4(4.2%) |  |
| Other | 1(2.5%) | 0(0%) | 2(2.5%) | 5(5.3%) |  |
| ***Surgical pathology Stage*** |  |  |  |  | **0.004** |
| I | 20(51.3%) | 3(37.5%) | 39(51.3%) | 26(28.3%) |  |
| II | 1(2.6%) | 1(12.5%) | 9(11.8%) | 24(26.1%) |  |
| III | 17(43.6%) | 4(50%) | 28(36.8%) | 36(39.1%) |  |
| IV | 1(2.6%) | 0(0%) | 0(0%) | 6(6.5%) |  |
| ***Tumour Grade*** |  |  |  |  | 0.377 |
| Low | 5(16.7%) | 1(12.5%) | 14(20.6%) | 12(14.1%) |  |
| Intermediate | 4(13.3%) | 2(25.0%) | 20(29.4%) | 16(18.8%) |  |
| High | 21(70.0%) | 5(62.5%) | 34(50.0%) | 57(67.1%) |  |
| ***Surgical Optimal Debulking*** |  |  |  |  | 0.141 |
| Optimally Debulked | 34(87.2%) | 8(100%) | 62(84.9%) | 64(75.3%) |  |
| Not Optimally Debulked | 5(12.8%) | 0(0%) | 11(15.1%) | 21(24.7%) |  |
| ***Platinum sensitivity*** |  |  |  |  | 0.285 |
| Sensitive | 36(94.7%) | 8(100%) | 63(96.9%) | 70(89.7%) |  |
| Resistant | 2(5.3%) | 0(0%) | 2(3.1%) | 8(10.3%) |  |

**Supplementary Table S6.** Multivariate analysis.

| **PFS** | | | | | | | | |
| --- | --- | --- | --- | --- | --- | --- | --- | --- |
|  | B | SE | Wald | df | Sig. | Exp(B) | 95.0% CI for Exp(B) | |
|  |  |  |  |  |  |  | Lower | Upper |
| **Platinum Sensitivity** | 3.662 | 0.489 | 56.109 | 1 | **0.0001** | 38.930 | 14.934 | 101.484 |
| **Polβ** | 0.626 | 0.249 | 6.328 | 1 | **0.012** | 1.870 | 1.148 | 3.046 |
| **PARG** | -0.555 | 0.284 | 3.824 | 1 | 0.051 | 0.574 | 0.329 | 1.001 |
| **OS** | | | | | | | | |
|  | B | SE | Wald | df | Sig. | Exp(B) | 95.0% CI for Exp(B) |  |
|  |  |  |  |  |  |  | Lower | Upper |
| **Platinum Sensitivity** | 2.525 | 0.352 | 51.457 | 1 | **0.0001** | 12.495 | 6.267 | 24.911 |
| **Polβ** | 0.367 | 0.215 | 2.921 | 1 | 0.087 | 1.444 | 0.948 | 2.199 |
| **PARG** | -0.468 | 0.245 | 3.639 | 1 | 0.056 | 0.626 | 0.387 | 1.013 |
|  |  |  |  |  |  |  |  |  |
