# Supplementary material for: Molecular disruption of DNA polymerase β for platinum sensitisation and synthetic lethality in epithelial ovarian cancers
Source: Oncogene. 2021 Mar 5;40(14):2496–508. doi: 10.1038/s41388-021-01710-y (PMC8032555; doi:10.1038/s41388-021-01710-y)
Supplement: Supplementary file 3 — Supplementary Figure legends [file 41388_2021_1710_MOESM3_ESM.docx]

**Supplementary figure legends:**

**Supplementary Figure S1:** **(A)** Polβ protein expression and Kaplan-Meier curve for overall survival in the whole cohort. Kaplan-Meier curve for *polβ* mRNA expression showing PFS in the test cohort **(B)**, validation cohort 1**(C)**, overall survival in test cohort **(D)** validation cohort 1 **(E)** and overall survival in validation cohort 2 (TCGA) **(F).**

**Supplementary Figure S2: (A)** Polβ nuclear and cytoplasmic extracts in A2780 and A2780cis treated with cisplatin Lysates collected 48h post treatment**. (B)** Validation of polβ antibody specificity by immunofluorescence in A2780 and A2780cis control and polβ knock out cells**. (C)** Representative photo micrographic images of polβ immunofluorescence staining in A2780 and A2780cis treated with cisplatin for 48h. **(D)** Quantification of polβ nuclear fluorescence by ImageJ software is shown here.

**Supplementary Figure S3:** Polβ mammalian interactome. See methods for details. Brown circle= query gene, blue circle= associated gene from same organism, yellow circle= associated gene from different organism, green circle= associated chemicals, green line + association with genetic evidence, yellow line = association with physical evidence, purple line = association with genetic and physical evidence, blue line = association with chemicals, greater note size represents increased connectivity and thicker edges sizes represent increased evidence supporting the association.

**Supplementary Figure S4: (A)** polβ knock down by SiRNA in A2780cis cells. **(B)** ATR levels by western blot in A2780cis control and polβ knock down at day3 & day5. **(C)** p-CHK1 levels by western blot in A2780cis control and polβ knock down at day3 & day5. **(D)** Validation of polβ knock down by siRNA construct2 in A2780cis cells. **(E)** Cisplatin sensitivity inA2780cis control and polβ knock down transfected with siRNA construct2. **(F)** polβ knock down in PE04 cells. **(G)** Polβ knock down in A2780 cells. **(H)** Validation of polβ knock down by siRNA construct2 in A2780 cells. **(I)** Cisplatin sensitivity by clonogenic survival assay inA2780 control and polβ knock down transfected with siRNA construct2.

**Supplementary Figure S5: (A)** Representative photo micrographic images of 53BP1 and γH2AX immunofluorescence staining in A2780 control and Polβ KO cells treated with cisplatin (5µM) for 24h. **(B)** Cisplatin sensitivity by clonogenic survival assay in PE01 (control), PE01(R) & PE01(R) polβ knock down. Polβ knock down in PE01R cells**. (C)** Polβ knock out in A2780cis cells. **(D)** Representative photo micrographic images of 53BP1 and γH2AX immunofluorescence staining in A2780cis control and Polβ KO cells treated with cisplatin (5µM) for 24h.

**Supplementary Figure S6: (A)** Cisplatin sensitivity by MTS cell proliferation assay in A2780 and A2780cis control and Polβ KO pre-treated with curcumin. Control and KO cells were plated overnight the following day A2780 Polβ KO cells were treated with 30 μM curcumin or left untreated. After 24h, cisplatin were added at the indicated doses. Cell proliferation were determined using MTS cell Titer reagent. **(B)** Mitomycin C sensitivity by MTS cell proliferation assay in A2780 and A2780cis control and Polβ KO cells. Representative images of flow cytometry analysis by weasel for γH2AX **(C)**, cell cycle **(D)** and apoptosis **(E).**

**Supplementary Figure S7: (A)** Representative photo micrographic images of invasion assay and quantification in A2780 and A2780cis control and Polβ_KD. **(B)** E-cadherin, N-cadherin, TGF-β and MMP-9 western blot in A2780 and A2780cis controls and Polβ_knockouts. **(C)** Protein levels quantification by western blotting for E-cadherin, N-cadherin, TGFβ and MMP-9 in A2780 and A2780cis control and Polβ_knockout. **(D)** E-cadherin, N-cadherin, TGF-β and MMP-9 western blot in A2780 and A2780cis controls and Polβ_knockouts.

**Supplementary Figure S8: (A)** Heat map showing EMT gene expression by RT2 profiler in A2780 control and A2780 Polβ_knockout cells. List of up-regulated and down-regulated genes is shown here. **(B)** Heat map showing EMT gene expression by RT2 profiler in A2780cis control and A2780cis Polβ_knockout cells. List of up-regulated and down-regulated genes is shown here.

**Supplementary Figure S9: (A)** Polβ & BRCA2 protein levels by western blot in PE01 and PE04 cells**. (B)** Pamoic acid sensitivity by clonogenic survival assay in A2780 and A2780cis control and polβ KO cells**. (C)** γH2AX analysis by flow cytometry for PEO1and PEO4 treated with NSC66719 (250 µM**). (D)** Cell cycle analysis by flow cytometry for PEO1 and PEO4 treated with NSC66719. **(E)** Apoptosis analysis by flow cytometry for PEO1and PEO4-treated with NSC66719. **(F)** Olaparib sensitivity in A2780 control and A2780 Polβ_KO. **(G)** Olaparib sensitivity in A2780cis control and A2780cis Polβ_KO. **(H)** PARGi sensitivity in PEO4 control and PEO4 Polβ_KD. **(I)** PARP1 quantification by western blot in A2780 control and A2780cis POLβ_KO.

**Supplementary Figure S10:** PARP1 levels by western blot in A2780 and A2780cis control and Polβ_KO cells**. (B)** Poly (ADP) ribose polymers levels by western blot in A2780 and A2780cis control and Polβ_KO cells**. (C) & (D)** Quantification of Poly (ADP) ribose polymers levels by western blot in A2780 and A2780cis control and Polβ_KO cells**. (E)** Poly (ADP) ribose polymers levels by western blot and quantification relative to housekeeper levels in A2780 and A2780cis control and Polβ_KO cells treated with Olaparib (10μM ) or PARGi (25 μM). (**F**) PAR quantification by ELISA. See results for details **(G)** Quantification of γH2AX positive cells by flow cytometry**. (H)** p-CHK1 levels by western blot in A2780 and A2780cis control and Polβ_KO cells treated with Olaparib (10μM) or PARGi (25 μM).

**Supplementary Figure S11: Clonogenic assays. (A)** Talazoparib sensitivity in A2780 control and A2780 Polβ_KO. **(B)** Talazoparib sensitivity in A2780cis control and A2780cis Polβ_KO.

**Supplementary Figure S12: (A)** PARG expression and Kaplan Meier curves for PFS in the whole cohort. **(B)** PARG expression and Kaplan Meier curves for overall survival in the whole cohort.
