# Supplementary figures and images for: Molecular disruption of DNA polymerase β for platinum sensitisation and synthetic lethality in epithelial ovarian cancers
Source: Oncogene. 2021 Mar 5;40(14):2496–508. doi: 10.1038/s41388-021-01710-y (PMC8032555; doi:10.1038/s41388-021-01710-y)

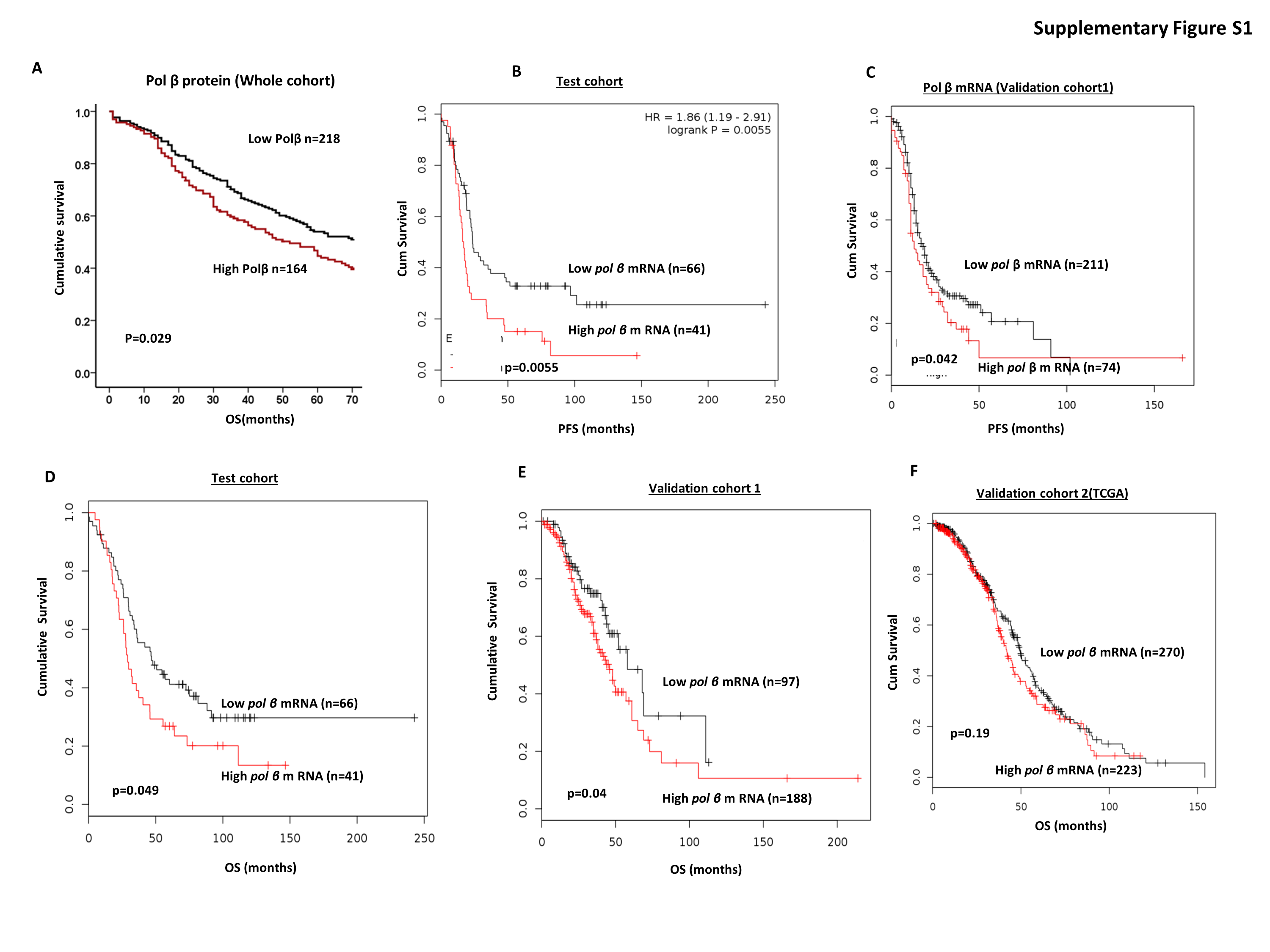

Supplement: Supplementary file 4 — Supplementary Figure S1 [file 41388_2021_1710_MOESM4_ESM.tif]

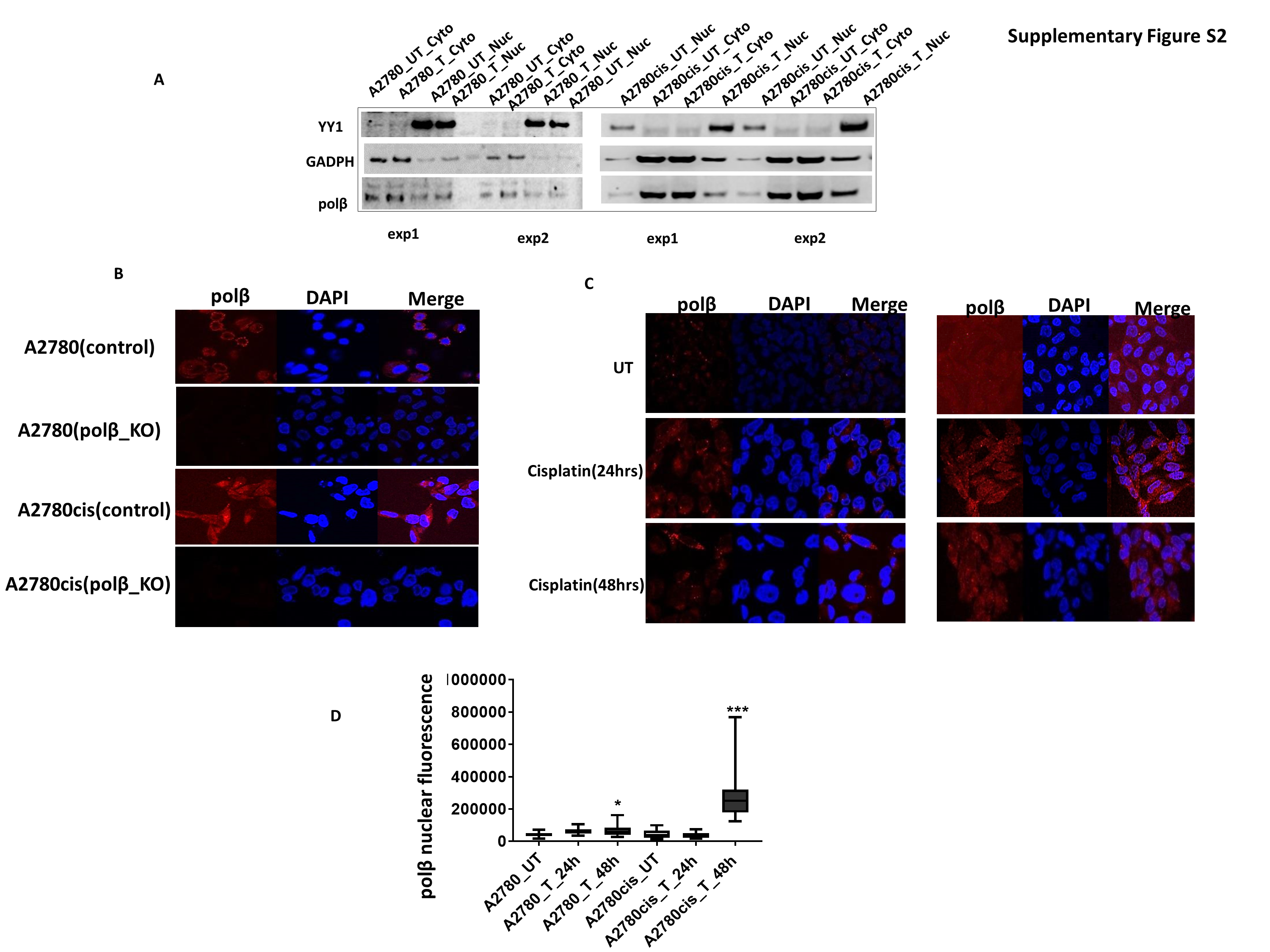

Supplement: Supplementary file 5 — Supplementary Figure S2 [file 41388_2021_1710_MOESM5_ESM.tif]

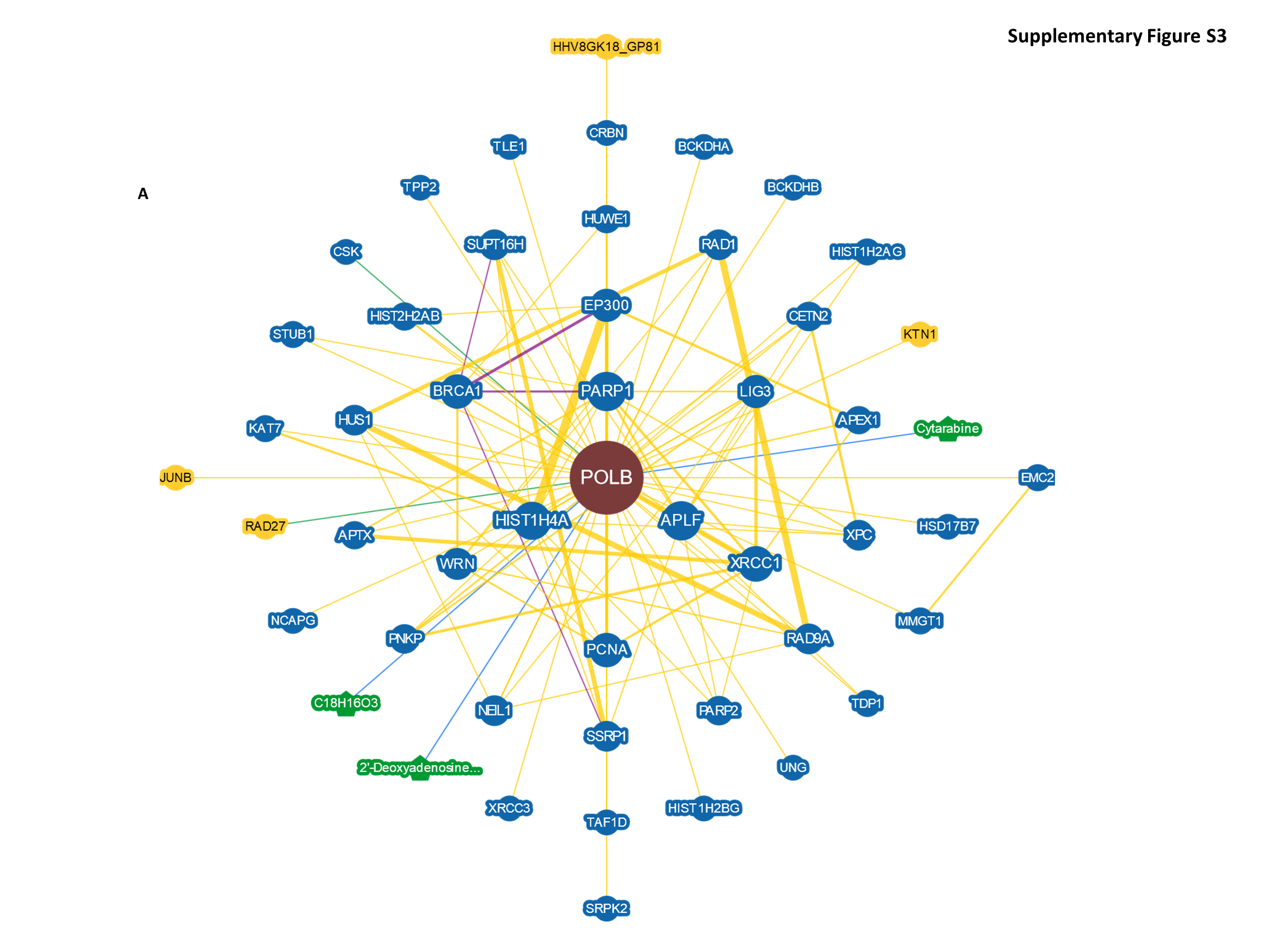

Supplement: Supplementary file 6 — Supplementary Figure S3 [file 41388_2021_1710_MOESM6_ESM.tif]

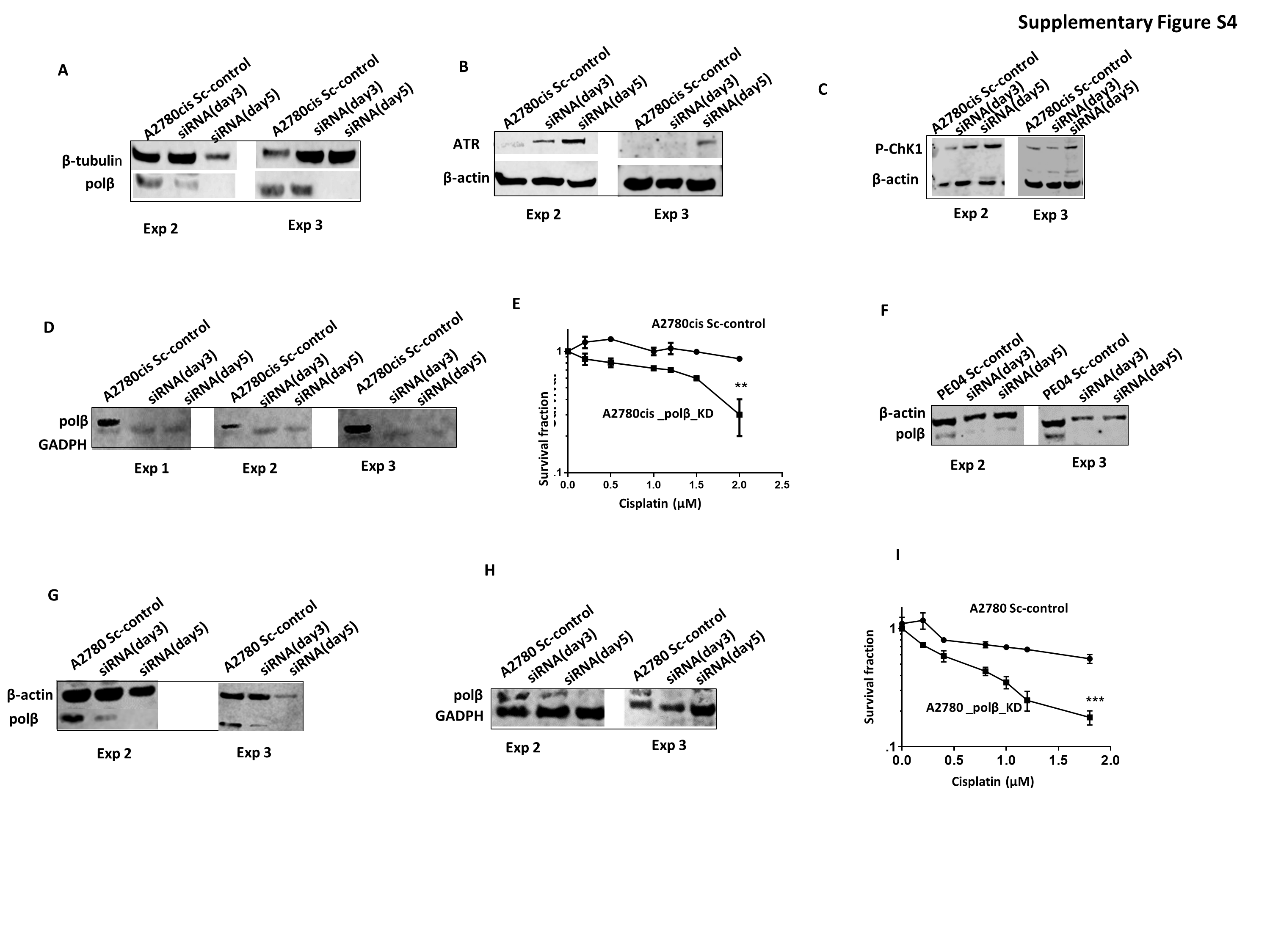

Supplement: Supplementary file 7 — Supplementary Figure S4 [file 41388_2021_1710_MOESM7_ESM.tif]

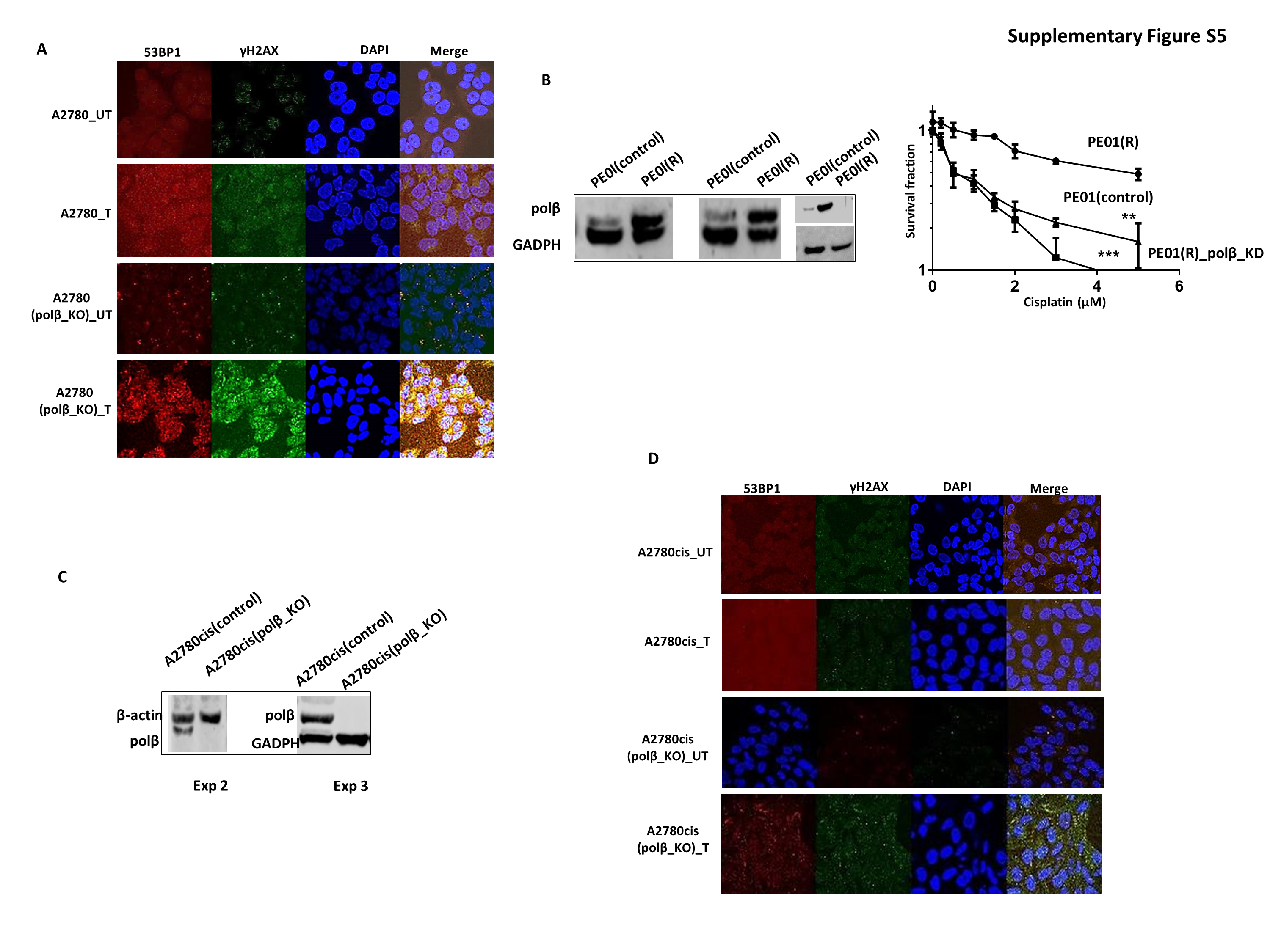

Supplement: Supplementary file 8 — Supplementary Figure S5 [file 41388_2021_1710_MOESM8_ESM.tif]

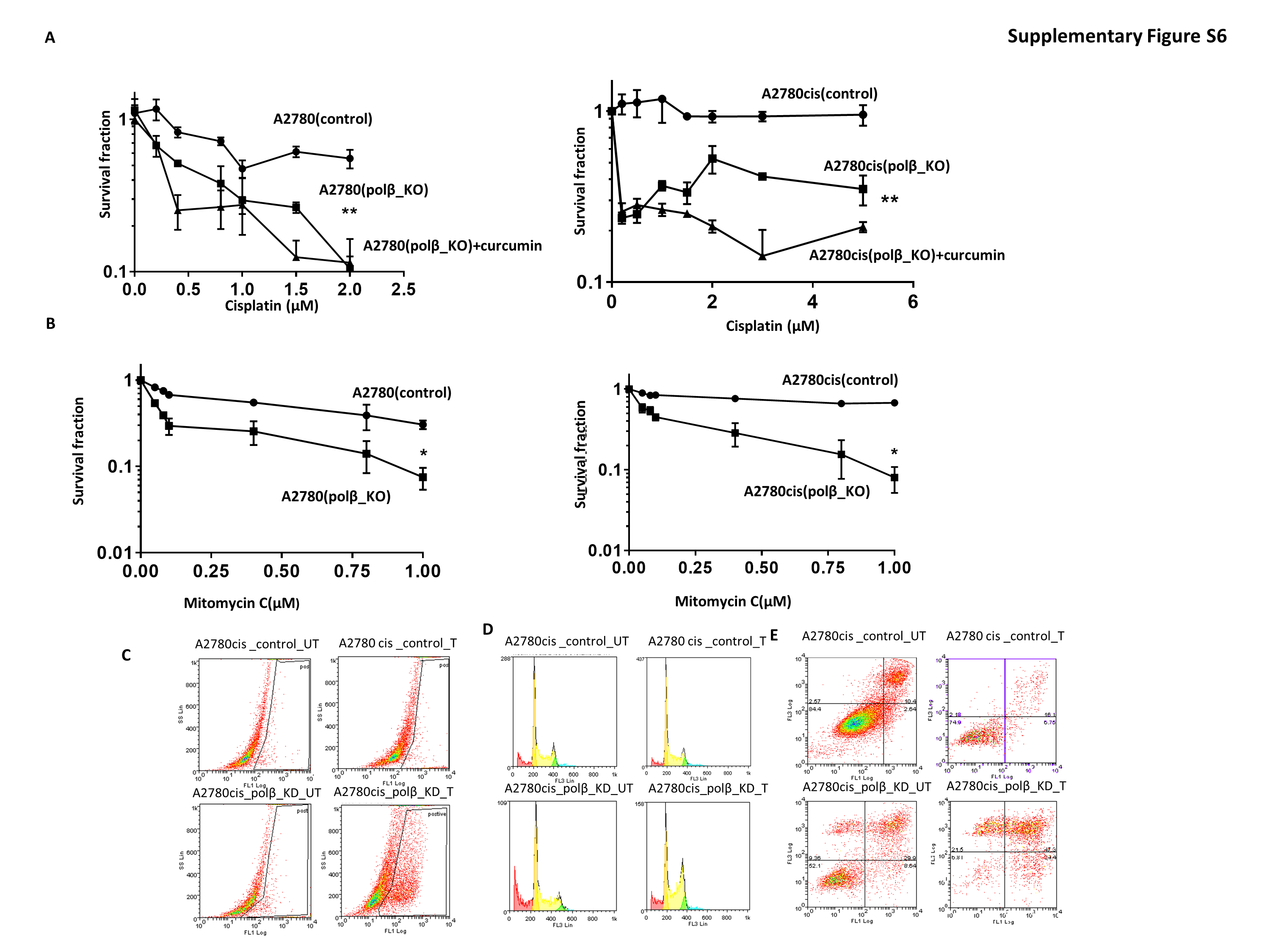

Supplement: Supplementary file 9 — Supplementary Figure S6 [file 41388_2021_1710_MOESM9_ESM.tif]

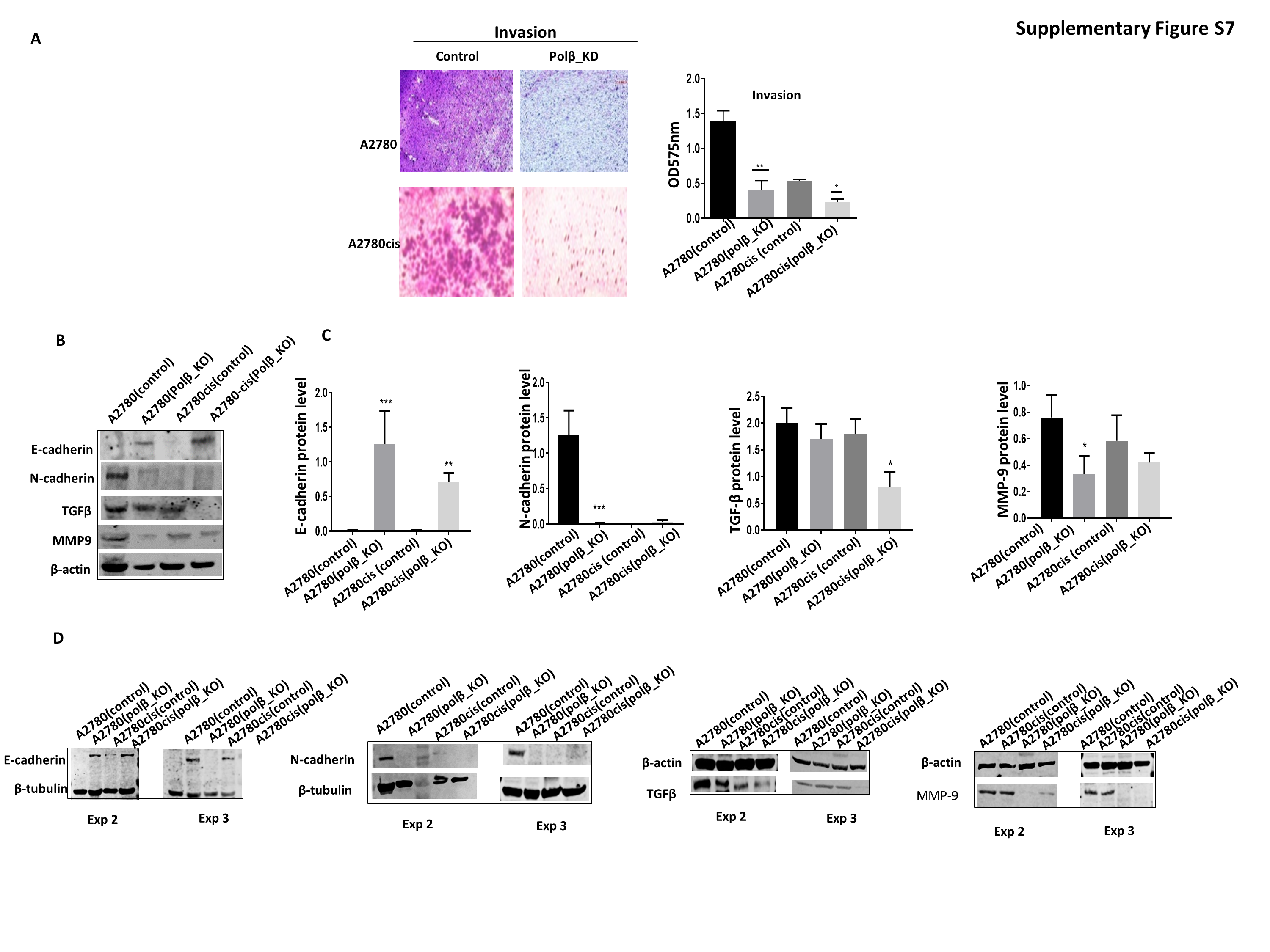

Supplement: Supplementary file 10 — Supplementary Figure S7 [file 41388_2021_1710_MOESM10_ESM.tif]

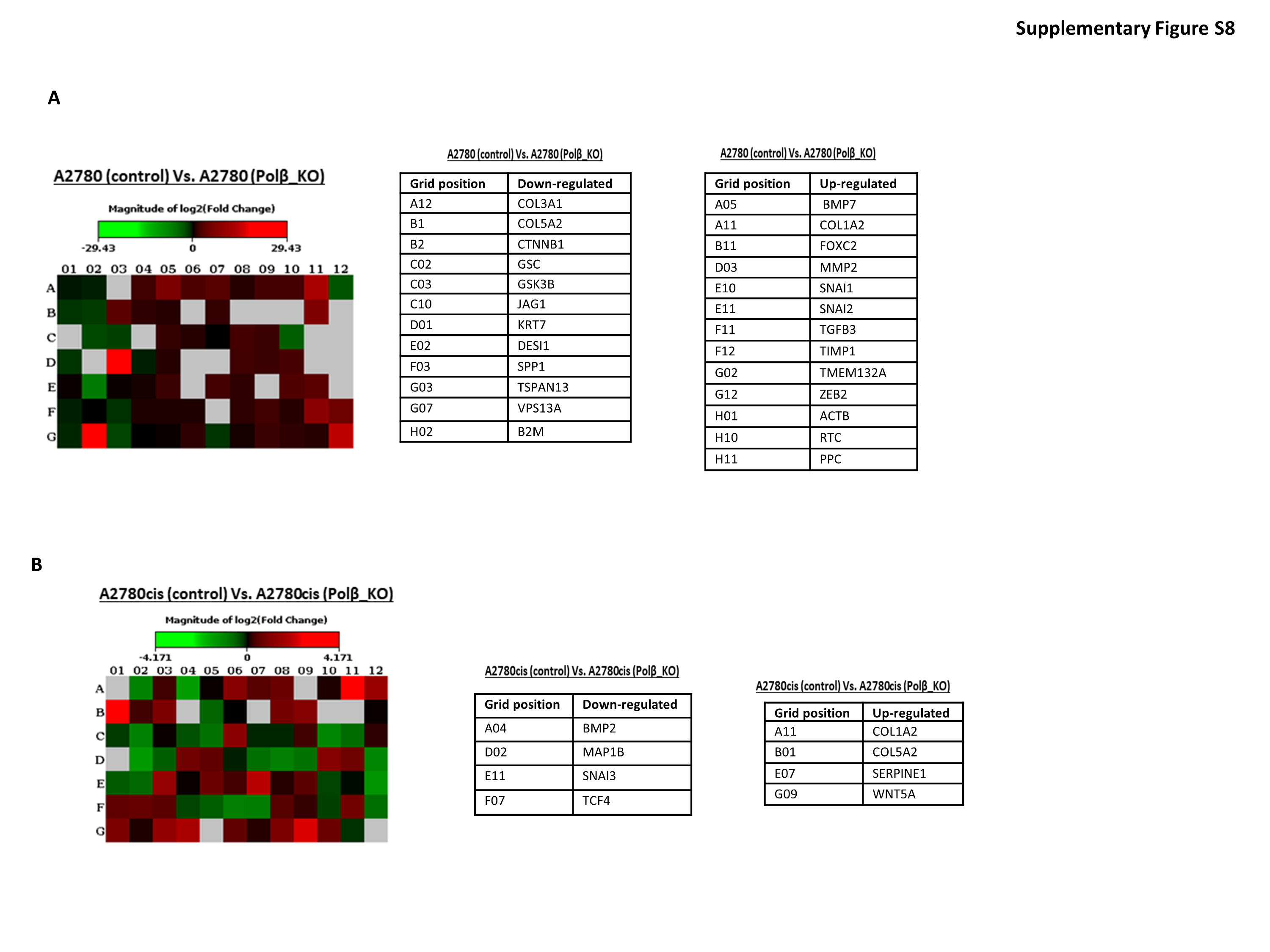

Supplement: Supplementary file 11 — Supplementary Figure S8 [file 41388_2021_1710_MOESM11_ESM.tif]

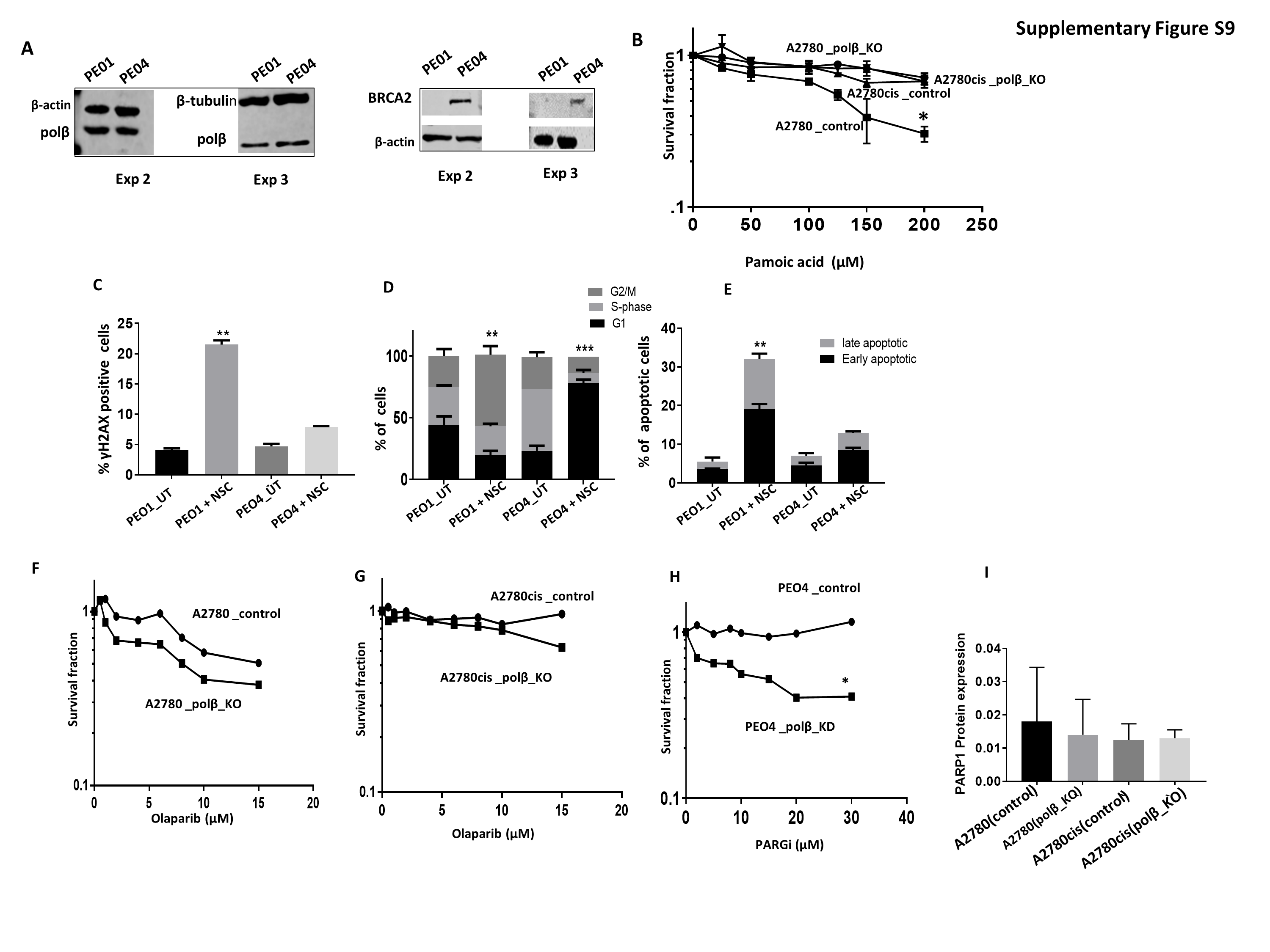

Supplement: Supplementary file 12 — Supplementary Figure S9 [file 41388_2021_1710_MOESM12_ESM.tif]

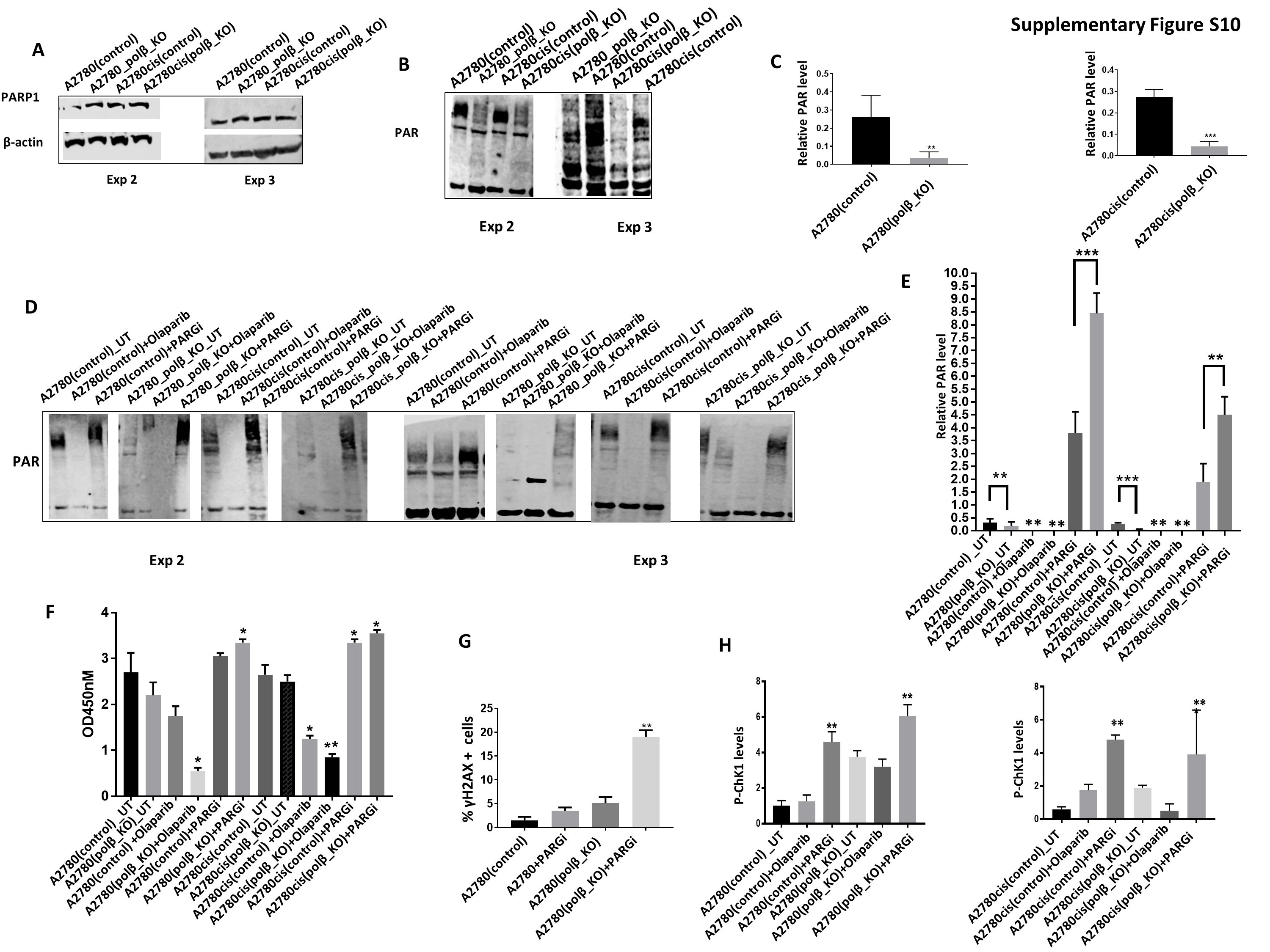

Supplement: Supplementary file 13 — Supplementary Figure S10 [file 41388_2021_1710_MOESM13_ESM.tif]

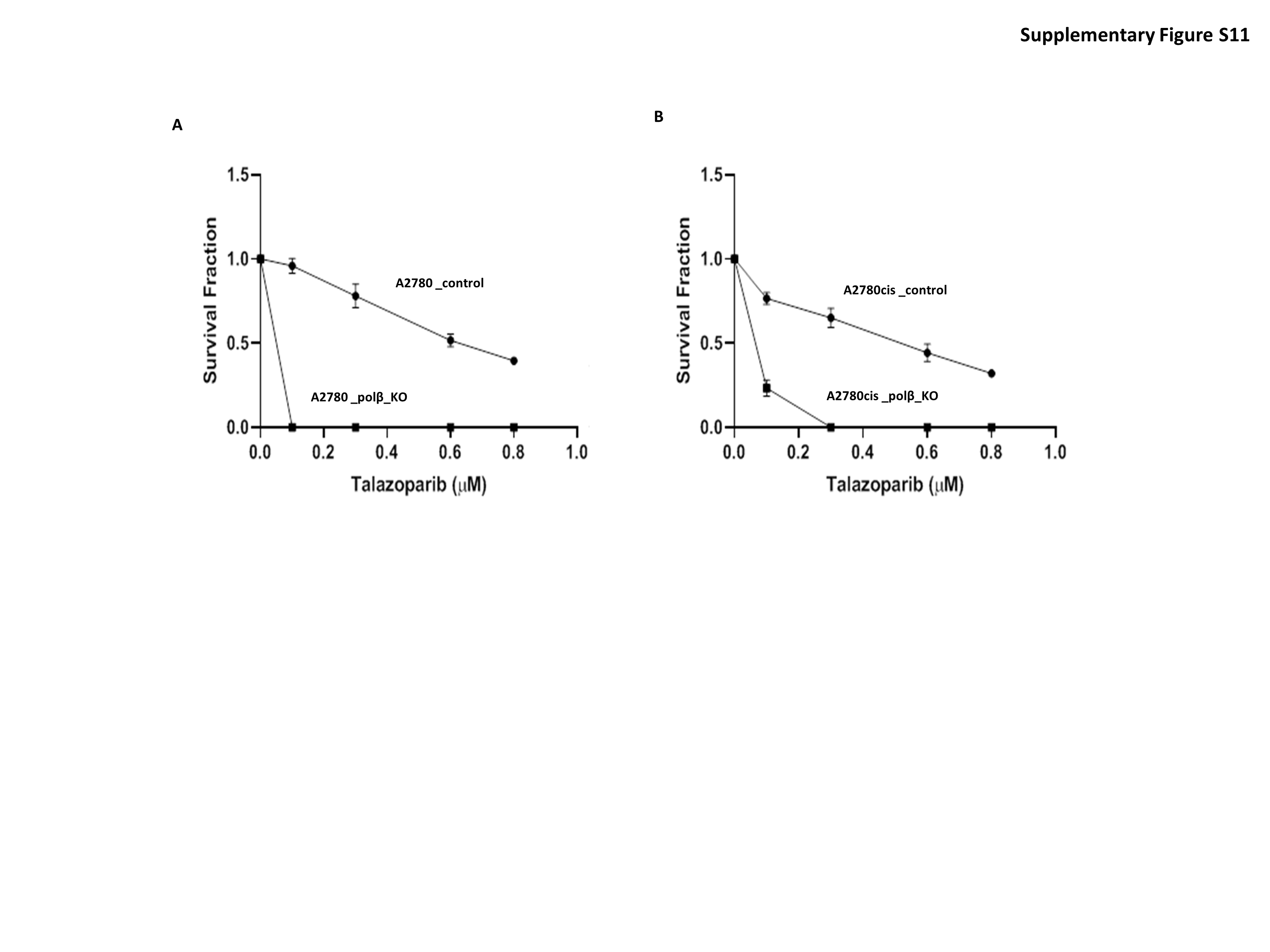

Supplement: Supplementary file 14 — Supplementary Figure S11 [file 41388_2021_1710_MOESM14_ESM.tif]

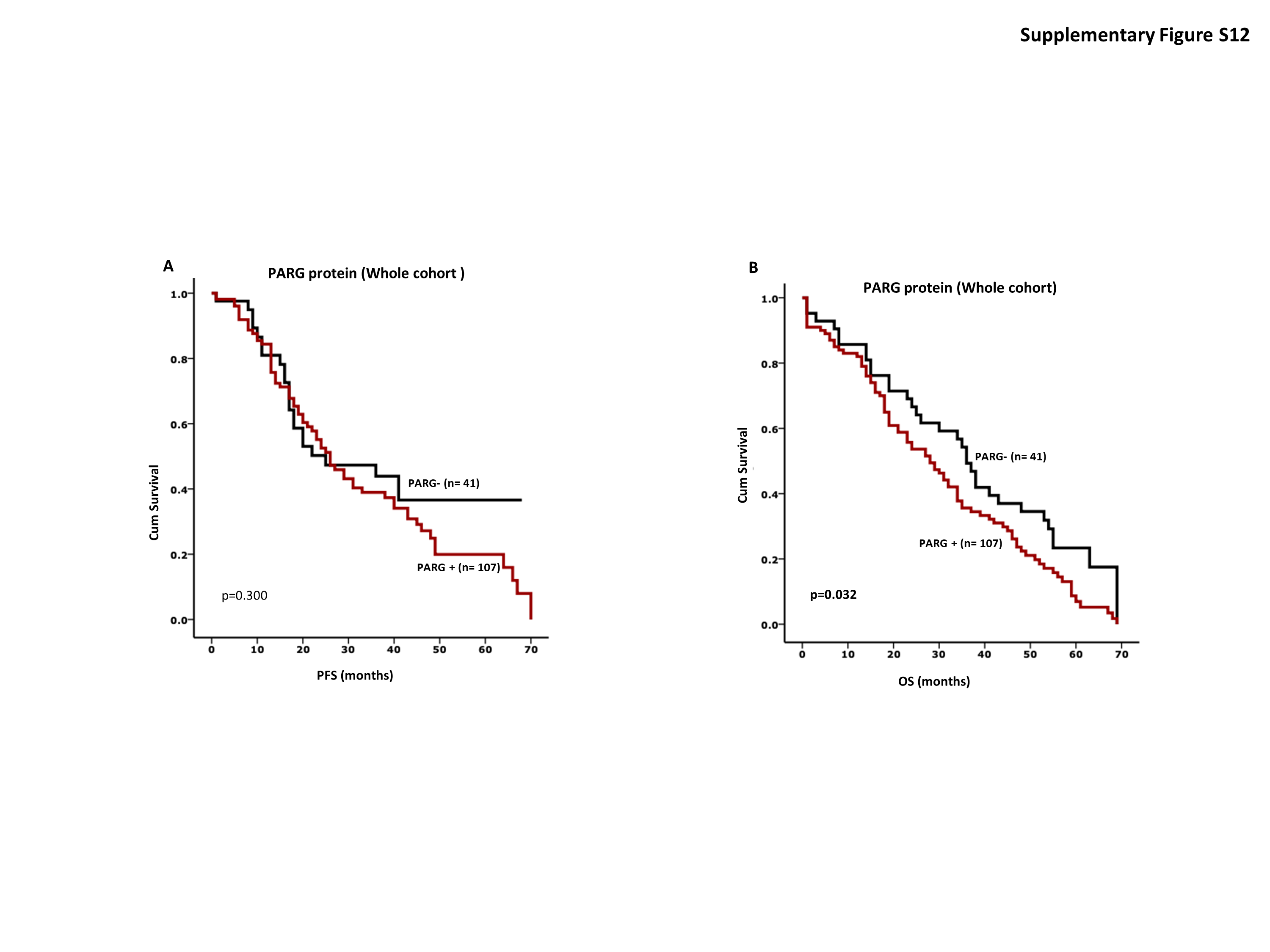

Supplement: Supplementary file 15 — Supplementary Figure S12 [file 41388_2021_1710_MOESM15_ESM.tif]
